# Investigating substituted phenylacetamide ligands in the D_4_R extended binding pocket

**DOI:** 10.1016/j.bmcl.2026.130660

**Published:** 2026-04-15

**Authors:** Tian Li, Mohammad Alkhatib, Peter A. Ramdhan, Lindsay Bourn, Jane K. Rainwater, Norman Nguyen, Caitlin P. Farrington, Blake Lewis, Chenglong Li, Thomas M. Keck, Comfort A. Boateng

**Affiliations:** a Department of Basic Pharmaceutical Sciences, Fred Wilson School of Pharmacy, High Point University, One University Parkway, High Point, NC 27268, United States; b Department of Chemistry & Biochemistry, Department of Biological & Biomedical Sciences, College of Science and Mathematics, Rowan University, 201 Mullica Hill Road, Glassboro, NJ 08028, United States; c Department of Medicinal Chemistry, University of Florida College of Pharmacy, 1345 Center Drive, Gainesville, FL 32610, United States

**Keywords:** Dopamine D_4_ receptor, Alkyl chain, Bicyclic, Phenylpiperazine, Molecular dynamics

## Abstract

Preclinical studies indicate that selective targeting of D_4_R may improve behavioral and cognitive outcomes in animal models relevant to cognitive disorders, like ADHD and Alzheimer's disease, and substance use disorders (SUDs). In this study, we extend upon prior development of analogs of A-412997, a D_4_R-selective partial agonist, by exploring ligand interactions within the secondary binding pocket. We modified the alkyl chain on the phenylacetamide's benzyl ring by extending and cyclizing the alkyl chain. A series of compounds were synthesized and tested using competition radioligand binding assays on D_2_R, D_3_R, and D_4_R, using [^3^H]*N*-methylspiperone as the competing radioligand. Herein, we report that while increasing the chain length beyond two carbons reduced D_4_R affinity and selectivity, cyclizing the alkyl chain enhanced D_4_R affinity and maintained selectivity over D_2_R and D_3_R by 120-fold or more. Molecular modeling results suggest that the cyclized rings engage in more favorable interactions within the D_4_R binding pocket.

Dopamine is a catecholaminergic neurotransmitter that acts *via* five receptor subtypes (D_1_R-D_5_R), all of which are G protein-coupled receptors (GPCRs) with seven transmembrane domains. Dopamine receptors are classified into two main subfamilies based on their signaling mechanisms and sequence homology: D_1_-like receptors (D_1_R and D_5_R) couple to Gα_s/olf_ protein subunits, while D_2_-like receptors (D_2_R, D_3_R, and D_4_R) couple to Gα_i/o_ protein subunits. The dopamine D_4_ receptor (D_4_R) is involved in a range of neurological and physiological functions, including cognition, motivation and reward, and emotion regulation.^1^ D_4_R has the lowest overall level of expression in the brain, but are predominantly expressed in the prefrontal cortex and mesolimbic system, including the hippocampus and amygdala. D_4_R signaling contributes to emotional regulation and behavior and plays a key role in cognition, attention, and executive function.^2,3^

The coding sequence of the human D_4_R gene (*DRD4*) exhibits extensive genetic polymorphism, particularly in its third exon in the region that encodes for the third intracellular loop (3IL) wherein a 48-bp variable number tandem repeat (VNTR) sequence repeats between two to 11 times.^4^ Genetic variations in *DRD4* are associated with neuropsychiatric disorders, such as Attention-Deficit/Hyperactivity Disorder (ADHD), substance use disorders (SUDs), and novelty-seeking behavior.^5-7^ Preclinical studies indicate that D_4_R activation alters behavioral and cognitive outcomes in animal models relevant to cognition and attention,^5^ with agonists improving performance in tasks like five-trial inhibitory avoidance task and novel object recognition.^8-12^ In animal models, D_4_R-selective antagonists reduced alcohol-taking and alcohol-seeking behaviors, and attenuated cocaine-seeking behavior in nonhuman primates.^13,14^

Despite its potential as a therapeutic target, D_4_R remains without a selective FDA-approved drug. A-412997 (compound **1**) is one of the most characterized D_4_R-selective ligands, acting as a partial agonist at the D_4_R.^2^ We recently developed several novel A-412997 analogs that retained D_4_R selectivity compounds and varied in signaling efficacy, with one subset of compounds featuring a replacement of the piperidine ring featured in A-412997 with piperazine (compound **2**) and then extending the methyl substituent on the benzene ring to an ethyl (compound **3**). The longer alkyl chain, which is expected to contribute to binding within a secondary binding pocket resulted in higher D_4_R affinity compared to the methyl analog, suggesting there could be space for further hydrophobic interactions.^2^ To further understand the contributions of this alkyl chain extension to the binding affinity and subtype selectivity, this structure–activity relationship (SAR) study compares a series of novel compounds with increasing alkyl chain lengths (from one to five carbons) with unconstrained structures as well benzocyclopentane, benzocyclohexane, and benzocycloheptane rings (Fig. 1).

The primary goal of this research study was to develop a library of new compounds that can expand our understanding of D_4_R receptor-ligand interactions. As shown in Fig. 1, compounds **2** and **3** were previously derived from the well-characterized compound **1** (A-412997), a high-affinity D_4_R partial agonist with moderate selectivity over D_2_R and D_3_R.^2^ Drawing on the prior observation that the increasing the alkyl chain of compound **3** resulted in improved D_4_R affinity over compound **2**,^2^ this study sought to more fully investigate hydrophobic interactions in the secondary binding pocket by developing a library with two main structural modifications: extending the *N*-(3-methylphenyl)acetamide moiety through sequential homologation of the alkyl chain and introducing cyclization.

Ligands were synthesized as outlined in Scheme 1 using routine *N*-alkylation reactions previously reported.^2,15,16^ The commercially available substituted aniline **10** was converted to the intermediate substituted phenyl-2-chloroacetamide by reacting with 2-chloroacetyl chloride in the presence of substituted aniline and methylene chloride at room temperature. This was followed by an alkylation reaction with commercially available 1-(pyridin-2-yl)piperazine in the presence of K_2_CO_3_ and NaI in CH_3_CN under reflux conditions to yield the desired target compounds **4**–**9**, respectively, with the exception of the synthesis of compounds **1**–**3** which was previously reported.^2^

To evaluate the effect of ethyl substitution on the phenyl ring, as shown in compound **3**, on binding affinity and selectivity, the ethyl group was replaced with propyl to obtain compound **4**, butyl to obtain compound **5**, and pentyl to obtain compound **6**. Furthermore, the contribution of cyclizing the alkyl chain was evaluated by cyclizing the propyl chain to form benzocyclopentane (indane) in compound **7**, the butyl chain to form benzocyclohexane (tetralin) in compound **8**, and the pentyl chain to form benzocycloheptane in compound **9**.

Binding data, as shown in Table 1, were measured and calculated by competing all ligands with D_2_-like antagonist radioligand, [^3^H]*N*-methylspiperone, in competition radioligand binding assays. As previously reported, substitution of the piperidine featured in **A-412997** with piperazine, creating compound **2**, significantly diminished affinity for D_2_R and D_3_R while only slightly reducing D_4_R binding affinity, resulting in a significant gain in D_4_R selectivity. As also previously reported,^2,18^ extending the alkyl chain of *N*-(3-methylphenyl)acetamide on the second pharmacophore from methyl to ethyl, resulting in compound **3**, slightly improved D_4_R affinity while maintaining selectivity over D_2_R and D_3_R. Within the novel compound library, elongation of the alkyl chain to propyl, butyl, and pentyl groups (compounds **4**, **5**, and **6**, respectively) successively reduced D_4_R affinity, with reduced subtype selectivity resulting primarily from the loss of D_4_R binding affinity.

Interestingly, cyclization of the alkyl chain into benzocyclopentane, benzocyclohexane, and benzocycloheptane (compounds **7**, **8**, and **9**, respectively) resulted in ligands with improved D_4_R affinity compared to **2** and **3**, with D_4_R receptor selectivity over D_2_R and D_3_R maintained. Compound **7** had the highest D_4_R affinity (7.9 ± 0.9 nM), while the larger compounds **8** and **9** exhibited binding affinities of 31.4 ± 6.6 nM and 25.9 ± 0.9 nM, respectively. At D_2_R, the binding affinity dropped upon ring expansion, from compound **7** (4020 ± 36 nM) to compounds **8** and **9** (> 10,000 nM). At D_3_R, compounds **7** (2300 ± 270 nM) and **9** (3100 ± 235 nM) had low affinity, while compound **8** had an affinity >10,000 nM. Overall, compounds **7** (benzocyclopentane), **8** (benzocyclohexane), and **9** (benzocycloheptane) maintained a D_4_R binding selectivity of ≥120-fold over D_2_R and D_3_R.

Central Nervous System (CNS) Multiparameter Optimization (MPO) scores for all ligands are also provided in Table 1; these scores estimate the probability of CNS exposure and blood-brain barrier (BBB) penetration, providing a preliminary assessment of the pharmacokinetic profiles and CNS desirability.^17^ Key physicochemical properties, including calculated logP (cLogP), calculated logD (cLogD), topological polar surface area (TPSA), molecular weight (MW), number of hydrogen bond donors (HBD), and the most basic pK_a_ center, were assessed as indicated in the supporting document. All compounds yielded CNS MPO scores ≥4, indicating favorable predicted CNS penetration profiles.

## Trajectory analysis

To evaluate how the alkyl substitutions altered ligand engagement in the D_4_R orthosteric binding pocket, molecular dynamics (MD) simulations were employed. The final 50 ns of each MD trajectory were analyzed to evaluate the stability of the ligand–receptor complexes following equilibration. Root mean square deviation (RMSD) of both the protein backbone and ligand heavy atoms was calculated across all frames (Figs. S1-S9). In all systems, RMSD values rapidly reached a plateau and remained stable throughout the remainder of the simulation, indicating that the complexes maintained consistent conformations during the analyzed time period. The absence of large RMSD fluctuations suggests that the ligand binding poses remained stable within the receptor binding site and that no major structural rearrangements occurred. These analyses confirm that the trajectories were sufficiently equilibrated and suitable for structural interpretation of ligand–receptor interactions.

## Binding pose analysis

**Fig. 2.**
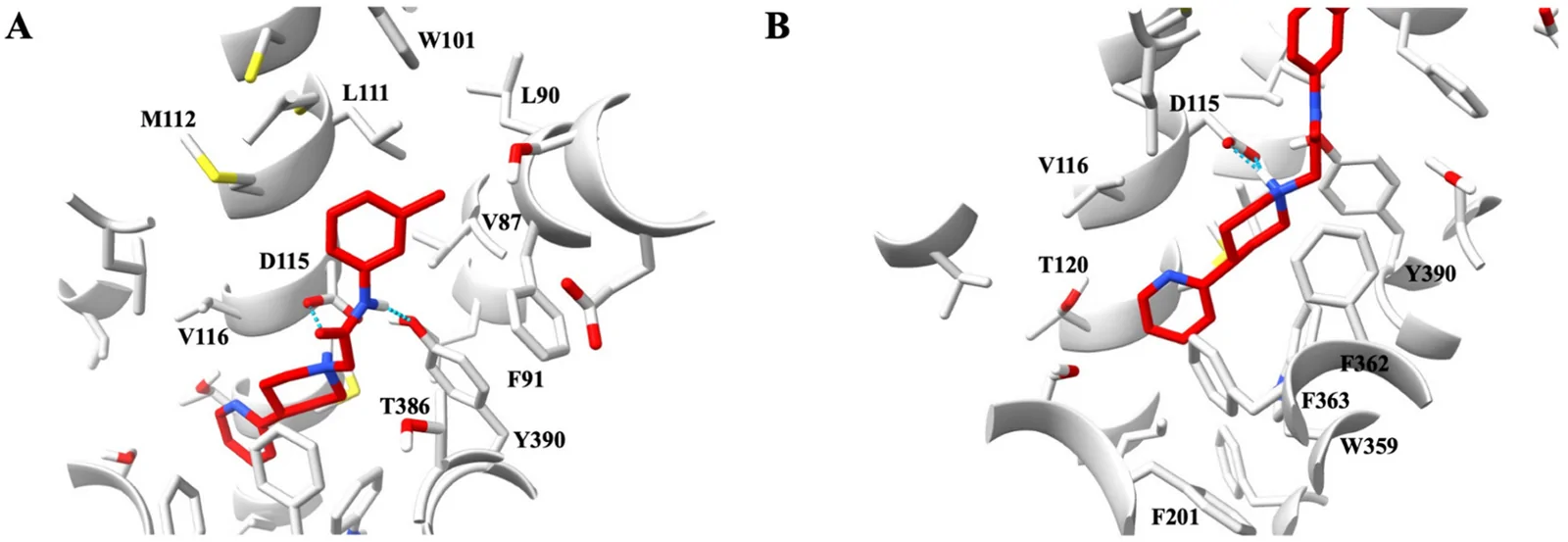
Image of the compound **1**-D_4_R Complex. **(A)** View of residues in the EBP that interact with Compound **1**. **(B)** View of residues in the OBP that interact with Compound **1**.

The top cluster from each MD simulation was used for binding pose analysis. After 50 ns, the binding site residues had adjusted to accommodate the ligand. Compound **1** was observed to fit well within the binding pocket, forming a hydrogen bond between its amide hydrogen and Y390, as well as maintaining the conserved interaction between its quaternary amine and D115 (Fig. 2). Within the orthosteric binding pocket (OBP), the pyridine ring primarily engages in hydrophobic interactions with residues V116, W359, F362, and F363. These OBP interactions remained largely consistent across all compounds. In the extended binding pocket (EBP), the methylphenyl ring of compound **1** is positioned within a hydrophobic cavity and interacts with residues V87, L90, F91, W101, L111, and M112.

In compound **2**, the introduction of a nitrogen adjacent to the pyridine ring shifted the hydrogen bonding preference of the amide hydrogen toward D115 rather than Y390. This interaction results in a slightly altered positioning of the methylphenyl group within the EBP (Fig. S10). In compound **3**, the extension of methylphenyl to an ethylphenyl group promotes hydrogen bonding between the amide hydrogen and Y390 in the top clustered model (Fig. S11). The ethylphenyl group remains embedded in the EBP, with the ethyl substituent oriented upward, making favorable hydrophobic contacts with residues F91, W101, and L111.

Compounds **4**–**6** explore the effect of alkyl chain length modifications on the phenyl ring. The introduction of a propylphenyl group in compound **4** causes a slight repositioning of the ligand within the binding pocket relative to compound **1** (Fig. S12). The propyl chain folds inward within the EBP, potentially disrupting the optimal orientation of the compound. The top clustered model indicates that the amide hydrogen engages D115 rather than Y390. Compound **5**, featuring a butyl substitution, adopts a pose similar to that of compound **4**. The amide hydrogen again interacts with D115, while the butyl chain extends outside the EBP, possibly engaging hydrophobic residues such as L90, F91, and W101 (Fig. S13). In compound **6**, the pentylphenyl modification results in a comparable binding pose. The pentyl chain extends into the EBP similarly to compound **4** (Fig. S14), and the amide hydrogen continues to favor interaction with D115. These observations suggest that increasing alkyl chain length may disrupt the compound's optimal positioning, which could contribute to the reduced binding affinity observed in experimental studies.

Compounds **7**–**9** investigate the effect of increasing ring size on the phenyl group to form a bicyclic moiety. In Compound **7**, the benzocyclopentane ring fits well within the EBP and engages in hydrophobic interactions with residues such as L90, F91, W101, and L111 (Fig. S15). The amide hydrogen forms a hydrogen bond with Y390, while the quaternary amine simultaneously engages D115. This suggests that the ring size supports a favorable orientation for dual hydrogen bonding and proper alignment of the bicyclic ring within the EBP. Compound **8**, containing a benzocyclohexane group, exhibits a similar binding mode (Fig. S16), again forming hydrogen bonds with both Y390 and D115 and positioning the ring deep within the EBP. Compound **9**, which incorporates a benzocycloheptane modification, follows the same interaction pattern (Fig. S17). Overall, these ring modifications appear to promote a conformation that simultaneously engages both key residues and optimally orients the ring within the EBP. This binding pose likely contributes to the improved binding affinities and selectivity observed for compounds **7**–**9**.

## Hydrogen bond analysis

**Fig. 3.**
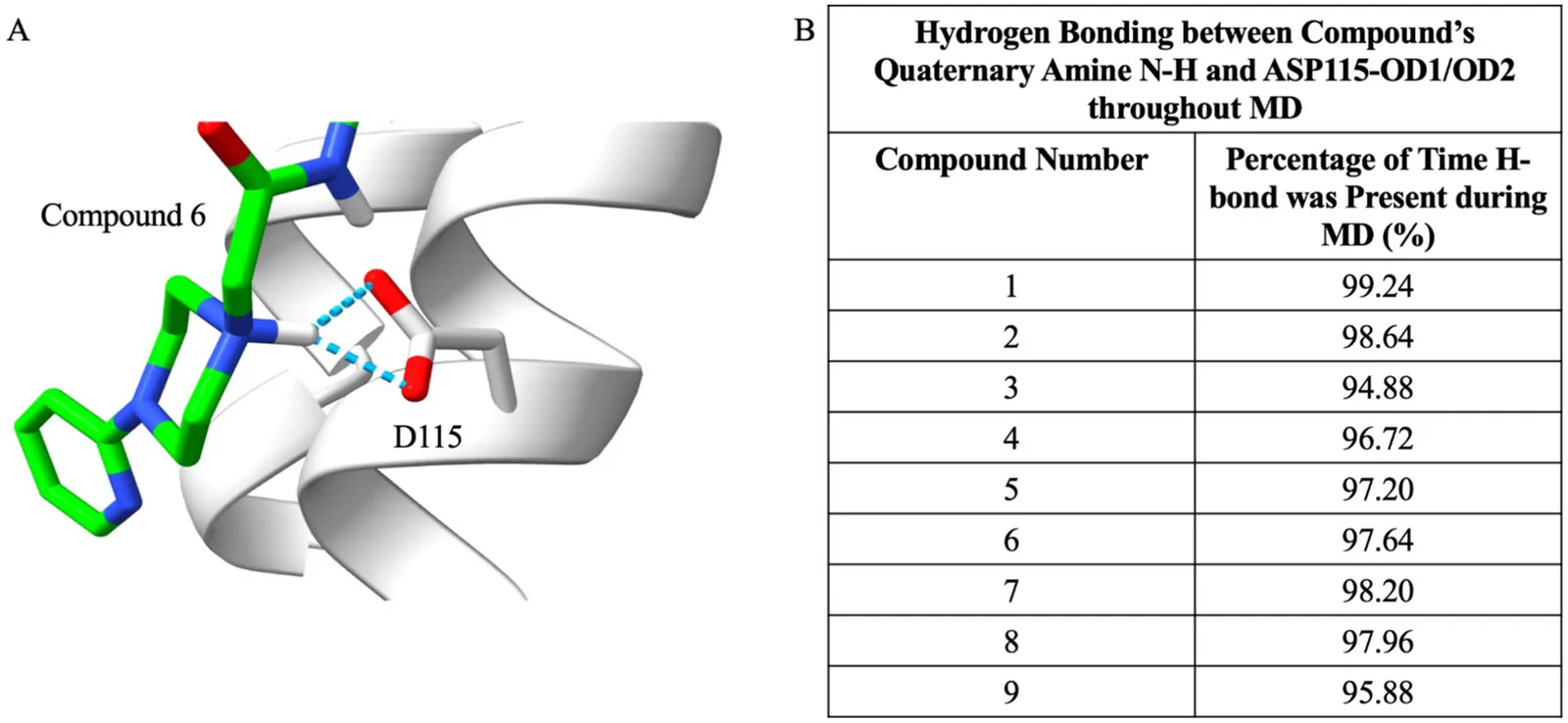
H-bond analysis of the compound's quaternary amine and D115 throughout the MD. **(A)** Image of the hydrogen bond being studied. **(B)** Percentage of the 50 ns MD where the quaternary amine formed a hydrogen bond with either oxygen of D115.

A hydrogen bond analysis was conducted to evaluate how the different chemical modifications influenced each compound's ability to interact with either D115 or Y390. We first examined whether the modifications affected the capacity of the quaternary amine to engage with either of the carboxylate oxygens of D115. Across the MD simulations, compounds **1** through **9** exhibited the following hydrogen bonding percentages: 99.24%, 98.64%, 94.88%, 96.72%, 97.20%, 97.64%, 98.20%, 97.96%, and 95.88%, respectively (Fig. 3). These results indicate that the modifications had minimal impact on the ability of the quaternary amine to maintain effective hydrogen bonding with D115.

**Fig. 4.**
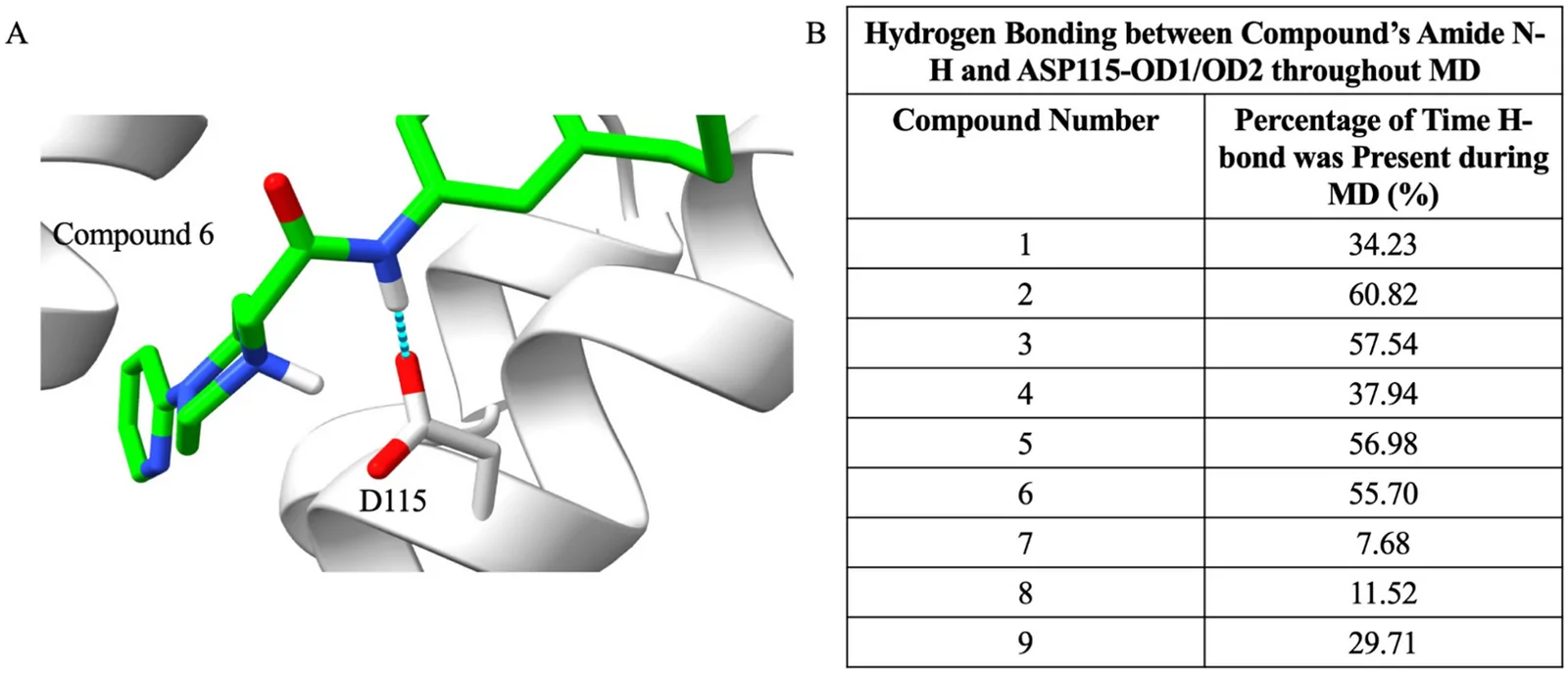
H-bond analysis of the compound's amide hydrogen and D115 throughout the MD. **(A)** Image of the hydrogen bond being studied. **(B)** Percentage of the 50 ns MD where the amide hydrogen formed a hydrogen bond with either oxygen of D115.

For the next analysis, we evaluated the ability of the compound's amide hydrogen to form hydrogen bonds with D115. In several simulations, the amide hydrogen formed hydrogen bonds with either D115 or Y390, depending on the structural modification present. The following hydrogen bonding percentages were observed for compounds **1** to **9**: 34.3%, 60.82%, 57.54%, 37.94%, 56.98%, 55.70%, 7.68%, 11.52%, and 29.71%, respectively (Fig. 4). Notably, the introduction of a nitrogen atom in Compounds **2** and **3** increased hydrogen bonding compared to Compound **1**. Compounds **4**, **5**, and **6** exhibited similar levels of hydrogen bonding, with slightly lower interaction in compound **4**. This suggests that alkyl chain length may influence hydrogen bonding, with longer chains potentially enhancing interactions. In contrast, ring modifications (Compounds **7**–**9**) resulted in significantly reduced hydrogen bonding, possibly due to conformational constraints that disfavor amide–D115 interactions. These observations may correlate with the increased selectivity associated with the ring-modified compounds.

**Fig. 5.**
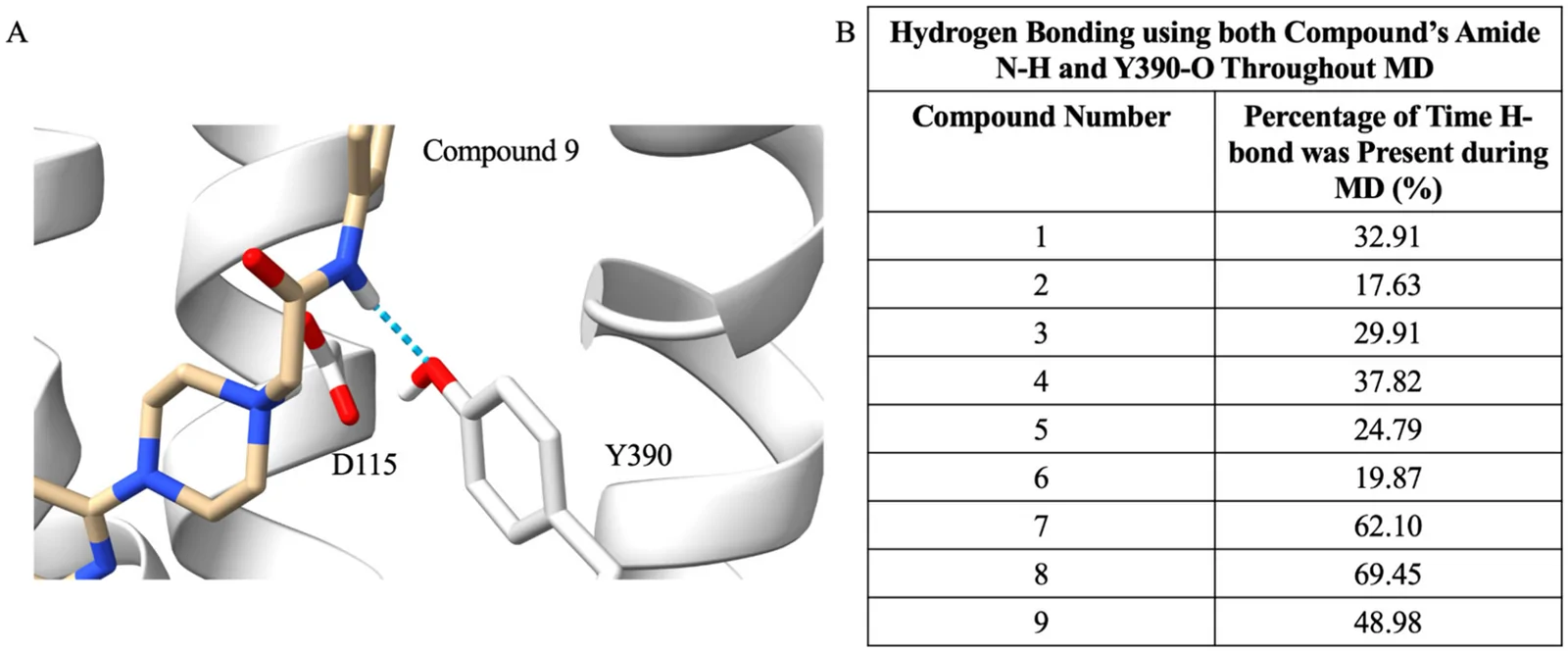
H-bond analysis of the compound's amide hydrogen and Y390 throughout the MD. **(A)** Image of the hydrogen bond being studied. **(B)** Percentage of the 50 ns MD where the amide hydrogen formed a hydrogen bond with Y390.

Next, we assessed the ability of compounds **1**–**9** to form hydrogen bonds between the amide hydrogen and Y390. The observed hydrogen bonding percentages for compounds **1**–**9** were: 32.91%, 17.61%, 29.91%, 37.82%, 24.79%, 19.87%, 62.10%, 69.45%, and 48.98%, respectively (Fig. 5). Compounds **1** through **6** exhibited this interaction in less than 40% of the simulation frames, whereas compounds **7** through **9** demonstrated markedly increased hydrogen bonding with Y390. These results suggest that the ring modifications present in compounds **7**–**9** favor an orientation that promotes amide–Y390 hydrogen bonding. This interaction may enhance the compound's ability to occupy the extended binding pocket (EBP), contributing to the increased selectivity observed for these analogs.

**Fig. 6.**
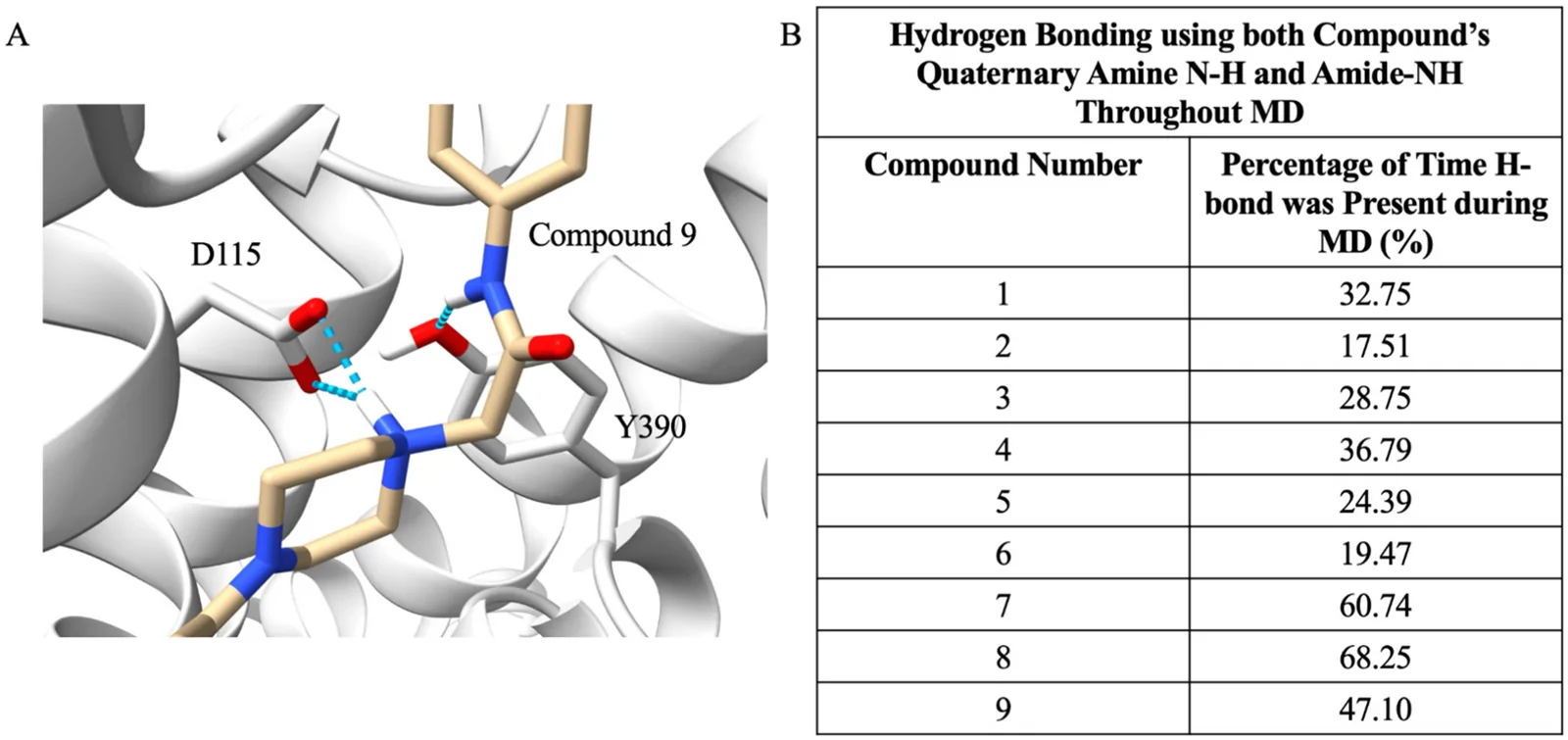
H-bond analysis of the combination of two hydrogen bonds: **(1)** the quaternary amine forming a hydrogen bond with either oxygen of D115 and **(2)** the amide hydrogen forming a hydrogen bond with Y390's oxygen. **(A)** Image of the hydrogen bonds being studied. **(B)** Percentage of the 50 ns MD where the hydrogen bonds are present.

Lastly, we evaluated the ability of compounds **1**–**9** to simultaneously form two key hydrogen bonds: **(1)** between the quaternary amine and either oxygen atom of D115, and **(2)** between the amide hydrogen and Y390. The corresponding percentages for compounds **1**–**9** were: 32.75%, 17.51%, 28.75%, 36.79%, 24.39%, 19.47%, 60.74%, 68.25%, and 47.10%, respectively (Fig. 6). This combined analysis was motivated by earlier observations suggesting a relationship between Y390 hydrogen bonding and increased selectivity. Compounds **1**–**6**, which exhibit lower selectivity, also show reduced engagement in these combined hydrogen bond interactions. In contrast, compounds **7**–**9** display substantially higher percentages, suggesting that these interactions promote an orientation favorable for engagement of the bicyclic ring with the extended binding pocket (EBP), thereby contributing to the enhanced selectivity observed for these analogs.

## Binding energy calculations

To evaluate how structural modifications influenced binding affinity, we employed both DeepAtom and Glide SP scoring methods. This comparison allowed us to assess how each tool captured key molecular interactions and contributed to the predicted binding energies. According to the DeepAtom model, compounds **1** through **9** yielded binding energies of −8.35, −8.11, −7.71, −8.37, −8.27, −8.85, −8.43, −9.02, and − 8.29 kcal/mol, respectively (Table 2). These results suggest that DeepAtom predicts relatively similar binding affinities across the compound series.

To complement the DeepAtom analysis, Glide SP scoring was used to evaluate binding energies across the compound series. Compared to DeepAtom, Glide SP produced slightly different values for compounds **1** through **9** (−8.51, −7.00, −7.75, −7.40, −8.46, −7.65, −8.41, −8.69, and −8.65 kcal/mol, respectively) (Table 2). Relative to compound **1**, compounds **2**, **3**, **4**, and **6** showed reduced binding scores, consistent with experimental binding data. Notably, compound **5**, which demonstrated weaker binding experimentally, received a Glide SP score comparable to compound **1**. This discrepancy may be due to additional hydrophobic contacts from the butyl side chain or the particular pose selected, which may have favored enhanced interactions in this scoring context. Compounds **7**, **8**, and **9** showed binding energies similar to compound **1**, aligning with experimental trends that indicate slightly stronger binding. Although small differences in binding scores were observed, the overall similarity suggests consistent pose quality and interaction profiles across the series.

Prior testing in animal models has shown that selectively activating D_4_R can enhance performance in tasks that challenge short-term memory, cognition, and attention. This suggests that D_4_R has potential as a therapeutic target for treating neuropsychiatric disorders associated with impaired cognition, such as ADHD, Alzheimer's disease, and the negative symptoms of schizophrenia. However, despite its therapeutic importance and potential in the treatment of these neuropsychiatric disorders, the clinical potential of D_4_R-targeted treatments remains limited by our understanding of ligand-receptor interactions.

In this study, we developed a small library of novel compounds to directly probe ligand interactions within the secondary binding pocket, focusing on the contributions of an alkyl chain of various lengths. The resulting compound library displayed a range of selectivity profiles across D_2_-like receptors. Longer free alkyl chains resulted in a loss of D_4_R affinity compared to ethyl analog **3**, but cyclized analogs resulted in improved D_4_R affinity. Notably, **7** exhibited the highest D_4_R affinity (K_i_ = 7.9 ± 0.9 nM), with 509-fold selectivity over D_2_R and 291-fold selectivity over D_3_R. *In silico* studies suggest that the high-affinity binding is driven by the ability of the bicyclic ring to form more extensive hydrophobic interactions within the extended binding pocket, maintaining a conformation that favors dual hydrogen bond interactions: an amide hydrogen with Y390 and the quaternary amine with D115. These new compounds offer promising tools for probing D_4_R pharmacology and investigating its role in neuropsychiatric disorders. Cyclized analogs **7**, **8**, and **9** also have CNS MPO scores multiparameter optimization (MPO) scores (≥ 4.2) thus representing promising new tools for medications development and better understanding D_4_R pharmacology and signaling mechanisms.

## Supplementary Material

Supplemental Information

## Figures and Tables

**Fig. 1.**
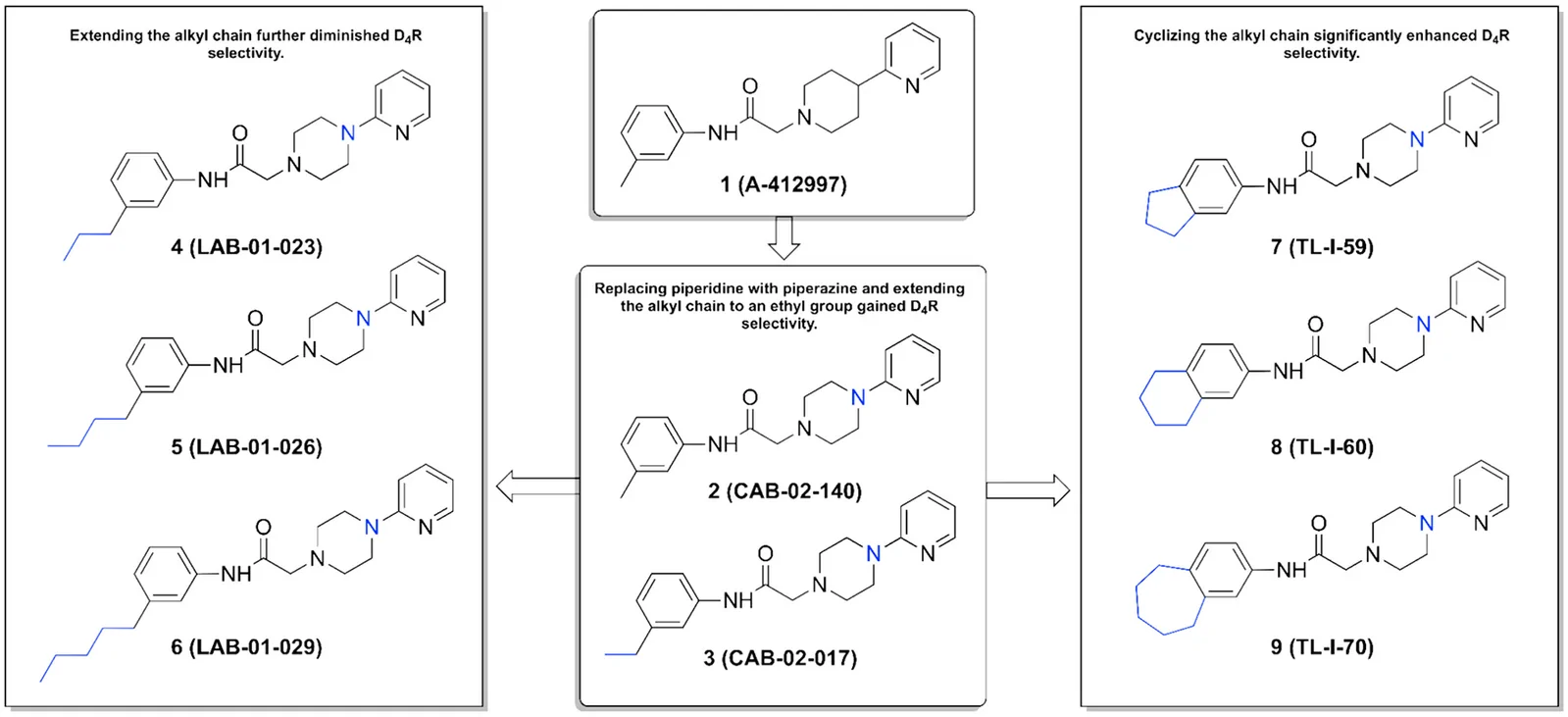
Alkyl chain length modification of compound **2** resulting in differing binding affinities at D_2_-like receptors.

**Scheme 1.**
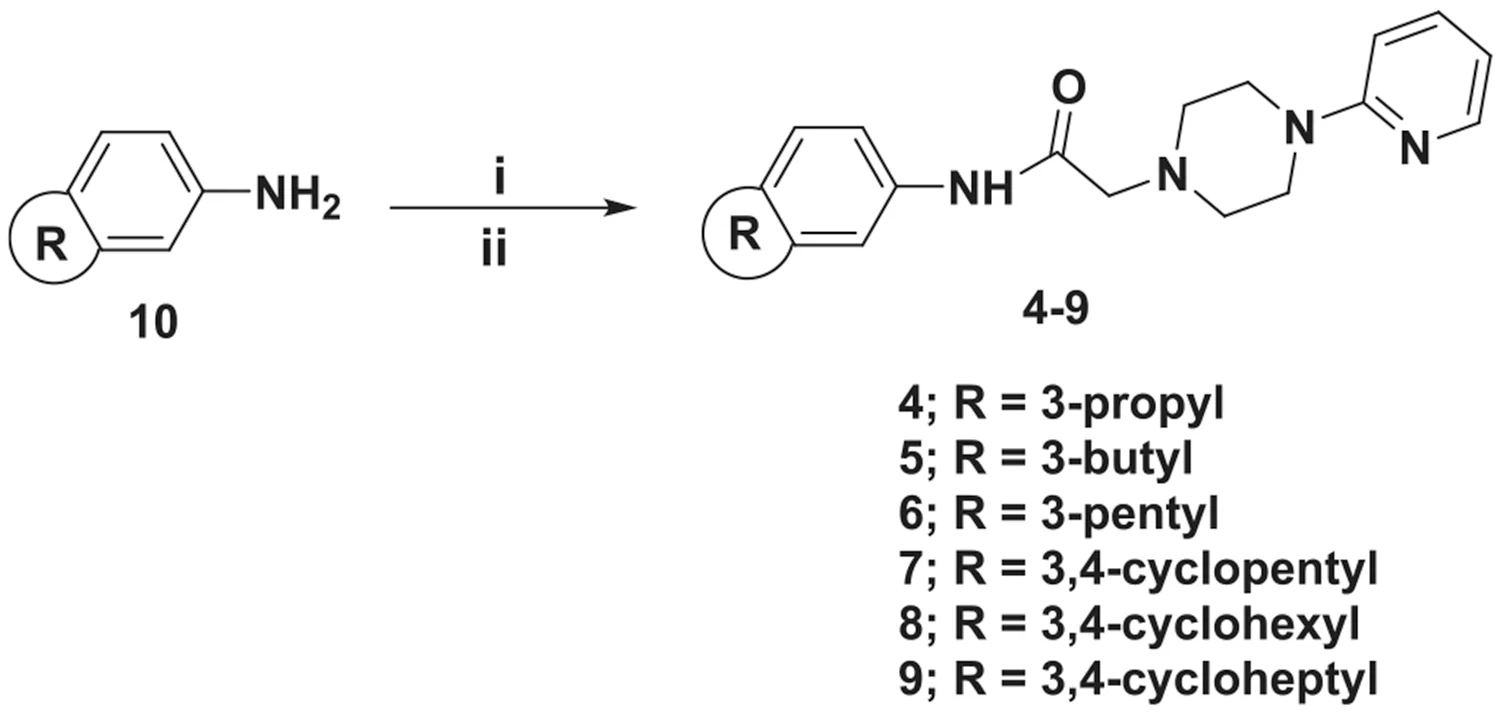
Synthesis of *N*-substitutedphenyl-2-(4-(pyridin-2-yl)piperazin-1-yl) acetamide compounds*^a^*. ^*a*^ Reagents and Conditions: (i) Substituted aniline, CH_2_Cl_2_, 0 °C to RT; (ii) CH_3_CN, NaI, K_2_CO_3_, Reflux, 1-(pyridin-2-yl)piperazine.

**Table 1:** Human dopamine D_2_-like receptor binding, cLogP, and Central Nervous System (CNS) Multiparameter Optimization (MPO) scores.^a^

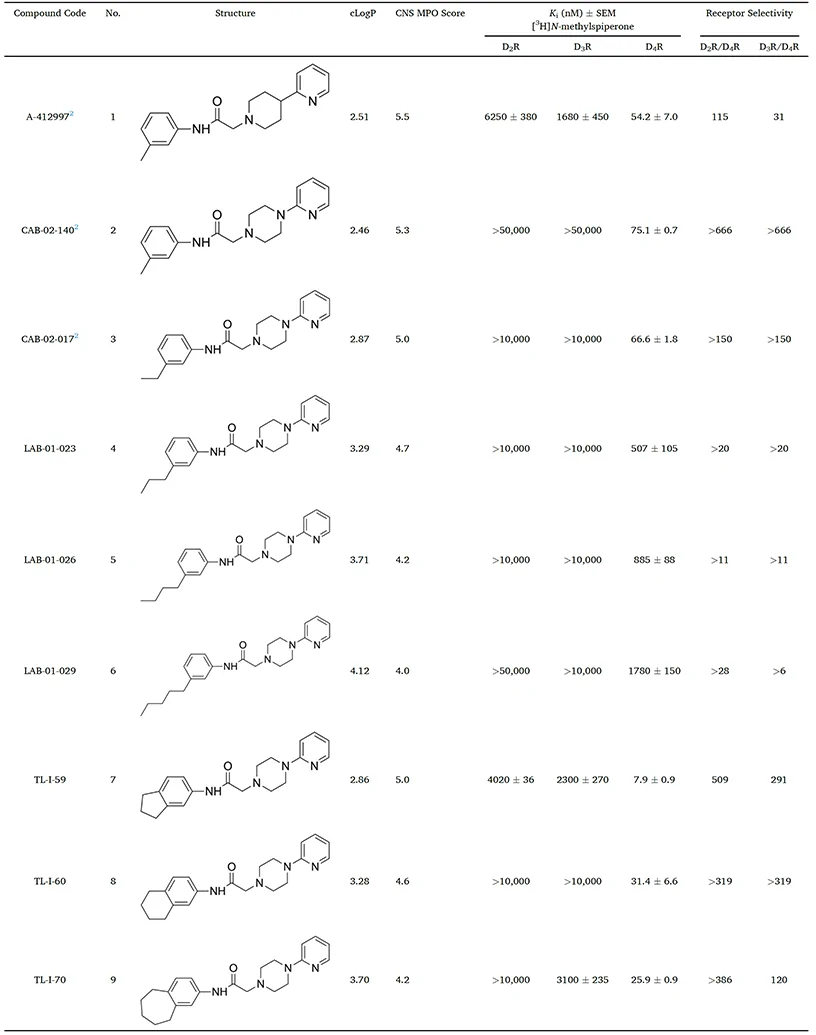

a *K*_i_ values determined by competitive inhibition of [^3^H]*N*-methylspiperone binding in membranes harvested from HEK 293 cells stably expressing hD_2_R, hD_3_R, or hD_4_R. All K_i_ values were determined from at least 3 independent experiments and are reported as means ± SEM. Pfizer's Central Nervous System (CNS) Multiparameter Optimization (MPO)^17^ scores are presented as a range from 0 to 6; higher CNS MPO scores (particularly those greater than 4) are more desirable for centrally active compound.

**Table 2:** Binding energies of compounds **1**–**9** bound to DRD4measured by the Deepatom Scoring Tool and Glide SP Scoring Tool.

Predicted Binding Energies of Compounds **1–9** bound to D4R (kcal/mol)		
Compound Number	DeepAtom	Glide SP
**1**	−8.35	−8.51
**2**	−8.11	−7.00
**3**	−7.71	−7.75
**4**	−8.37	−7.40
**5**	−8.27	−8.46
**6**	−8.85	−7.65
**7**	−8.43	−8.41
**8**	−9.02	−8.69
**9**	−8.29	−8.65

## Data Availability

No data was used for the research described in the article.

## Appendix A. Supplementary data

Supplementary data to this article can be found online at https://doi.org/10.1016/j.bmcl.2026.130660.

## Abbreviations:

TM: transmembrane
D_2_R: dopamine D_2_ receptor
D_3_R: dopamine D_3_ receptor
D_4_R: dopamine D_4_ receptor
CD_2_Cl_2_: deuterated dichloromethane
EtOAc: Ethyl acetate
PP: phenylpiperazine
