# Supplementary material for: Investigating substituted phenylacetamide ligands in the D4R extended binding pocket
Source: Bioorg Med Chem Lett. Author manuscript; Available in PMC 2026 Sep 15. (PMC13575890; doi:10.1016/j.bmcl.2026.130660)
Supplement: Supplementary file 1 [file NIHMS2197434-supplement-Supplemental_Information.pdf]

Supporting information

Investigating Substituted Phenylacetamide Ligands in the D_4R Extended Binding Pocket

Tian Li, [a,#] *Mohammad Alkhatib,*[b,#] *Peter A. Ramdhan,*[c,#] *Lindsay Bourn,*[a] *Jane K. Rainwater,* [a] *Norman Nguyen,*[b] *Caitlin P. Farrington,* [a] *Blake Lewis,* [a] *Chenglong Li,*[c] *Thomas M. Keck,*[b,*] *Comfort A. Boateng* [a,*]

[a]Department of Basic Pharmaceutical Sciences, Fred Wilson School of Pharmacy, High Point University, One University Parkway, High Point, North Carolina 27268, United States

[b]Department of Chemistry & Biochemistry, Department of Biological & Biomedical Sciences, College of Science and Mathematics, Rowan University, 201 Mullica Hill Road, Glassboro, New Jersey 08028, United States

[c]Department of Medicinal Chemistry, University of Florida College of Pharmacy, 1345 Center Drive, Gainesville, Florida 32610, United States

[#] Equally Contributing Authors, * Corresponding Authors

Content **Page**

Content	Page
**Experimental methods**	**S-3 - S-14**
**Figures S1-S9. RMSD values from the compounds- DRD4 MD simulation**	**S-15 - S-23**
**Figures S10-S17. Image of the compounds- DRD4 Complex interaction.**	**S-24 - S-26**
**Table S1. Physicochemical Properties of the Compounds**	**S-27**
**Table S2. for CNS MPO value and method of calculation of compound 5**	**S-28**
**Table S3. CNS (MPO) Scores of compounds 1-9**	**S-29**
**Table S4. Microanalysis data of compounds 4-9**	**S-30**
**Figures S18. HPLC traces of compound 9.**	**S-31**
**Figure S19-S24. ^{1}H and ^{13}C NMR spectra of compounds 4-9**	**S-32 – S-37**
**References**	**S-38 – S-40**

Experimental Methods

Synthesis.

Reaction conditions and yields were not optimized. Anhydrous solvents were purchased from Sigma Aldrich and were used without further purification. All other chemicals and reagents were purchased from Enamine, VWR Chemical BDH, TCI America, and Thermo Scientific Chemicals. All amide final products were converted into hydrochloride salt, except for compound **9** which was tested in free base form. Spectroscopic data and yields refer to the free base form of compounds. Flash chromatography was performed using silica gel (SiliCycle Inc.; 230-400 mesh, 60 Å) by using a Teledyne ISCO CombiFlashRF system. All amine final products were converted into either oxalate or hydrochloride salt. Spectroscopic data and yields refer to the free base form of compounds. Flash chromatography was performed using silica gel (EMD Chemicals, Inc.; 230-400 mesh, 60 Å) by using a Teledyne ISCO CombiFlash RF system. ^{1}H and ^{13}C spectra were acquired using a JEOL ECZ-400S NMR spectrometer. All ^{1}H and ^{13}C NMR experiments are reported in δ units and were measured relative to the signals for CD_2Cl_2 (δ_H 5.32 ppm and δ_C 53.84 ppm). Chemical shifts, multiplicities, and coupling constants (J) have been reported and calculated using MNova 64. Combustion elemental analysis was performed by Atlantic Microlab, Inc. (Norcross, GA) and the results agree within ± 0.4% of calculated values **(Table S2)**. cLogP values were calculated using ChemDraw version 23.1.1 The CNS-MPO scores were calculated using ChemDraw, version 23.0 and ChemAxon Marvin version.[1] The purity of compound **9** was determined using HPLC (Shimadzu Scientific Instruments Incorporated; LC-40 XR). The HPLC separation was achieved using a Kinetix C_{18} column (100mm×3mm i.d.,1.7µm, Phenomenex Inc.) equilibrated at 45 °C and a flow rate of 0.7 mL/min. The mobile phase comprised a linear gradient of CH_3CN/H_2O with 0.1% HCOOH starting at 15% CH_3CN to 100% over 20 min. The HPLC-

PDA chromatogram of compound **9** was used to measure its purity. Melting point determinations were conducted using an SRS OptiMelt MPA100-Automated melting point apparatus and are uncorrected. Based on NMR and combustion elemental analysis data, all final compounds are ≥ 95% pure. Compounds **1-3** have been previously described in the peer-reviewed literature.[1]

General Method.[2]

The intermediate substituted phenyl-2-chloroacetamide was obtained by stirring a solution of substituted-aniline (1 equiv.) in CH_2Cl_2 (30 mL) at 0 °C with added triethylamine (1.1 equiv.). Chloroacetyl chloride (1.05 equiv.) was slowly added to the reaction mixture. The reaction mixture was allowed to stir at 0 °C, monitored by TLC (50:50 EtOAc:Hexane). The reaction was quenched with water (50 mL) once it reached completion, then the mixture was extracted with CH_2Cl_2 (50 mL) 3 times. The organic layer was combined, washed with saturated NaCl solution, and dried over sodium sulfate. The combined organic layer was dried under reduced pressure to give crude product; the crude product was then purified by column chromatography to yield the final product. The alkylation reaction was performed by stirring a solution of substituted phenyl-2-chloroacetamide (1 equiv.) in CH_3CN (10 mL) was added to a mixture of sodium iodide dihydrate (0.1 equiv.), followed by potassium carbonate (1.2 equiv.) and 1-(2-pyridyl) piperazine (1 equiv.). The reaction mixture was stirred at 80 °C, monitored by TLC (100% EtOAc). The reaction was quenched using water (50 mL) once it reached completion, and the CH_3CN was evaporated under reduced pressure. The dried residue was extracted with CH_2Cl_2 (50 mL) 3 times. The organic layer was combined, washed with saturated NaCl solution, and dried over sodium sulfate. The combined organic layer was dried under reduced pressure to give crude product; the crude product was purified by column chromatography to yield the final product.

***N*-(3-propylphenyl)-2-(4-(pyridin-2-yl)piperazin-1-yl)acetamide (4)**. The compound was synthesized using 3-propylaniline by following the General Method. The product was purified by flash column chromatography (100 % EtOAc) to yield a white solid (743 mg, 93%). ^{1}H NMR (400 MHz, CD_2Cl_2) δ 9.10 (s, 1H), 8.16 (d, *J* = 4.6 Hz, 1H), 7.50 (ddd, *J* = 9.0, 7.2, 2.0 Hz, 1H), 7.42 (d, *J* = 8.1 Hz, 1H), 7.37 (s, 1H), 7.23 (t, *J* = 7.8 Hz, 1H), 6.94 (d, *J* = 7.6 Hz, 1H), 6.68 (d, *J* = 8.6 Hz, 1H), 6.64 (dd, *J* = 12.0, 6.3 Hz, 1H), 3.61 (t, *J* = 9.8, 5.1 Hz, 4H), 3.15 (s, 2H), 2.72 (t, *J* = 9.8, 5.1 Hz, 4H), 2.57 (t, *J* = 7.8 Hz, 2H), 1.63 (td, *J* = 37.4, 30.0, 15.2, 7.4 Hz, 2H), 0.94 (dd, *J* = 14.6, 7.3 Hz, 3H). ^{13}C NMR (101 MHz, CD_2Cl_2) δ 168.33, 159.77, 148.25, 144.23, 138.17, 137.78, 129.09, 124.61, 119.76, 117.12, 113.80, 107.39, 62.50, 53.65 (2C), 45.72 (2C), 38.33, 25.01, 13.98. The HCl salt was precipitated from ethanol. MP: 91.8 - 94.2 °C. Anal. ($C_{20}H_{26}N_4O$•2HCl•H_2O) C, H, N.

***N*-(3-butylphenyl)-2-(4-(pyridin-2-yl)piperazin-1-yl)acetamide (5).** The compound was synthesized using 3-butylaniline by following the General Method. The product was purified by flash column chromatography (100 % EtOAc) to yield a white solid (714 mg, 91%). ^{1}H NMR (400 MHz, CD_2Cl_2) δ 9.10 (s, 1H), 8.16 (d, *J* = 4.6 Hz, 1H), 7.50 (ddd, *J* = 8.8, 7.1, 2.0 Hz, 1H), 7.41 (d, *J* = 8.1 Hz, 1H), 7.37 (s, 1H), 7.23 (t, *J* = 7.8 Hz, 1H), 6.94 (d, *J* = 7.5 Hz, 1H), 6.69 (d, *J* = 8.6 Hz, 1H), 6.64 (dd, *J* = 12.1, 5.0 Hz, 1H), 3.61 (t, *J* = 5.0 Hz, 4H), 3.15 (s, 2H), 2.72 (t, *J* = 5.0 Hz, 4H), 2.64 – 2.56 (m, 2H), 1.64 – 1.55 (m, 2H), 1.35 (td, *J* = 29.9, 7.3 Hz, 2H), 0.92 (t, *J* = 7.4 Hz, 3H). ^{13}C NMR (101 MHz, CD_2Cl_2) δ 168.34, 159.77, 148.25, 144.47, 138.17, 137.79, 129.10, 124.57, 119.71, 117.07, 113.81, 107.40, 62.50, 53.66 (2C), 45.73 (2C), 35.98, 34.07, 22.78, 14.10. The HCl salt was precipitated from ethanol. MP: 74.5 – 75.0 °C. Anal. ($C_{21}H_{28}N_4O$•2HCl•1.25H_2O) C, H, N.

***N*-(3-pentylphenyl)-2-(4-(pyridin-2-yl)piperazin-1-yl)acetamide (6).** The compound was synthesized using 3-pentylaniline by following the General Method. The product was purified by flash column chromatography (100 % EtOAc) to yield a white solid (523 mg, 95%). ^{1}H NMR (400 MHz, CD_2Cl_2) δ 9.10 (s, 1H), 8.16 (d, *J* = 4.5 Hz, 1H), 7.50 (ddd, *J* = 8.4, 7.0, 1.9 Hz, 1H), 7.41 (d, *J* = 8.1 Hz, 1H), 7.37 (s, 1H), 7.23 (t, *J* = 7.8 Hz, 1H), 6.94 (d, *J* = 7.6 Hz, 1H), 6.68 (d, *J* = 8.6 Hz, 1H), 6.64 (dd, *J* = 7.1, 5.0 Hz, 1H), 3.61 (t, *J* = 5.0 Hz, 4H), 3.15 (s, 2H), 2.72 (t, *J* = 5.1 Hz, 4H), 2.59 (t, *J* = 7.8 Hz, 2H), 1.61 (p, *J* = 7.7 Hz, 2H), 1.39 – 1.29 (m, 4H), 0.89 (t, *J* = 6.6 Hz, 3H). ^{13}C NMR (101 MHz, CD_2Cl_2) δ 168.32, 159.76, 148.25, 144.50, 138.18, 137.78, 129.10, 124.55, 119.71, 117.08, 113.80, 107.39, 62.50, 53.65 (2C), 45.71 (2C), 36.26, 31.91, 31.61, 22.92, 14.22. The HCl salt was precipitated from ethanol. MP: 67.8 – 68.9 °C. Anal. ($C_{22}H_{30}N_4O{\bullet}2HCl{\bullet}H_2O$) C, H, N.

***N*-(2,3-dihydro-1*H*-inden-5-yl)-2-(4-(pyridin-2-yl)piperazin-1-yl)acetamide (7).** The compound was synthesized using 2,3-dihydro-1*H*-inden-5-amine by following the General Method. The product was purified by flash column chromatography (100 % EtOAc) to yield a white solid (520.5 mg, 97%). ^{1}H NMR (400 MHz, CD_2Cl_2) δ 9.07 (s, 1H), 8.16 (ddd, *J* = 5.1, 2.1, 1.1 Hz, 1H), 7.55 – 7.45 (m, 1H), 7.49 (s, 1H), 7.26 (d, *J* = 8.1 Hz, 1H), 7.16 (d, *J* = 8.0 Hz, 1H), 6.68 (d, *J* = 8.5 Hz, 1H), 6.64 (dd, *J* = 7.2, 5.0 Hz, 1H), 3.60 (t, *J* = 5.1 Hz, 4H), 3.14 (s, 2H), 2.88 (dt, *J* = 20.8, 7.4 Hz, 4H), 2.71 (t, *J* = 4.8 Hz, 4H), 2.04 (tt, *J* = 22.2, 7.7 Hz, 2H). ^{13}C NMR (101 MHz, CD_2Cl_2) δ 168.15, 159.73, 148.22, 145.54, 140.42, 137.76, 136.37, 124.71, 117.85, 116.10, 113.76, 107.35, 62.46, 53.62 (2C), 45.69 (2C), 33.34, 32.65, 25.99. The HCl salt was precipitated from ethanol. MP: 161.7 °C. Anal. ($C_{20}H_{28}Cl_2N_4O_2$) C, H, N.

2-(4-(pyridin-2-yl)piperazin-1-yl)-*N*-(5,6,7,8-tetrahydronaphthalen-2-yl)acetamide (8). The compound was synthesized using 2,3-dihydro-1*H*-inden-5-amine by following the General

Method. The product was purified by flash column chromatography (100 % EtOAc) to yield a white solid (460.8 mg, 82%). ^{1}H NMR (400 MHz, CD_2Cl_2) δ 9.01 (s, 1H), 8.16 (ddd, *J* = 5.0, 2.0, 1.0 Hz, 1H), 7.50 (ddd, *J* = 8.8, 7.1, 2.0 Hz, 1H), 7.29 – 7.24 (m, 1H), 7.25 (s, 1H), 7.01 (d, *J* = 8.0 Hz, 1H), 6.68 (d, 1H), 6.63 (dd, *J* = 7.2, 5.0 Hz, 1H), 3.60 (t, *J* = 5.0 Hz, 4H), 3.13 (s, 2H), 2.78 – 2.67 (m, 4H), 2.70 (t, *J* = 5.0 Hz, 4H), 1.77 (p, *J* = 3.4 Hz, 4H). ^{13}C NMR (101 MHz, CD_2Cl_2) δ 168.14, 159.73, 148.22, 138.13, 137.75, 135.55, 133.44, 129.72, 120.11, 117.31, 113.75, 107.35, 62.45, 53.61 (2C), 45.68 (2C), 29.84, 29.19, 23.65, 23.51. The HCl salt was precipitated from ethanol. MP: 154.8 °C. Anal. ($C_{21}H_{30}Cl_2N_4O_2$) C, H, N.

2-(4-(pyridin-2-yl)piperazin-1-yl)-*N*-(6,7,8,9-tetrahydro-5*H*-benzo[7]annulen-2-yl)acetamide (9). The compound was synthesized using 6,7,8,9-tetrahydro-5*H*-benzo[7]annulen-2-amine by following General Method. The product was purified by flash column chromatography (100 % EtOAc) to yield a white solid (559.1 mg, 96%). ^{1}H NMR (400 MHz, CD_2Cl_2) δ 9.04 (s, 1H), 8.16 (ddd, *J* = 4.9, 2.0, 0.9 Hz, 1H), 7.50 (ddd, 1H), 7.32 (dd, *J* = 8.0, 2.3 Hz, 1H), 7.27 (d, *J* = 2.3 Hz, 1H), 7.05 (d, *J* = 8.0 Hz, 1H), 6.68 (dd, *J* = 8.6, 0.9 Hz, 1H), 6.64 (ddd, *J* = 7.1, 4.9, 0.8 Hz, 1H), 3.64 – 3.57 (m, 4H), 3.14 (s, 2H), 2.76 (td, 4H), 2.71 (t, 4H), 1.83 (tt, *J* = 5.9, 5.9 Hz, 4H), 1.68 – 1.55 (m, 2H). ^{13}C NMR (101 MHz, CD_2Cl_2) δ 168.16, 159.78, 148.26, 144.70, 139.94, 137.77, 135.98, 129.73, 120.47, 117.11, 113.78, 107.38, 62.49, 53.66 (2C), 45.73 (2C), 37.00, 36.37, 33.03, 28.97, 28.81. The compound was kept in free base form. MP: 158.9-159.1 °C. Compound purity greater than 95% was confirmed with HPLC.

Radioligand binding assays.

Binding at dopamine D_2-like receptors was determined similarly to previously described methods.[3, 4] Membranes were prepared from HEK293 cells stably expressing human $D_{2L}R$, D_3R, or D_4R grown in a 50:50 mix of DMEM and Ham's F12 culture media, supplemented with 20 mM HEPES, 2 mM L-glutamine, 0.1 mM non-essential amino acids, 1X antibiotic/antimycotic, 10% heat-inactivated fetal bovine serum, and 200 μg/mL hygromycin (Life Technologies, Grand Island, NY) and kept in an incubator at 37 °C and 5% CO_2. Upon reaching 80-90% confluence, cells were harvested using pre-mixed Earle's Balanced Salt Solution (EBSS) with 5 mM EDTA (Life Technologies) and centrifuged at 2,000 rpm for 10 min at 4 °C. The supernatant was removed, and the pellet was resuspended in 10 mL hypotonic lysis buffer (5 mM $MgCl_2 \cdot 6\ H_2O$, 5 mM Tris, pH 7.4 at 4 °C) and centrifuged at 15,800 rpm (~25,000 g) for 30 min at 4 °C. The pellet was then resuspended in fresh EBSS binding buffer made from 8.7 g/L Earle's Balanced Salts without phenol red (US Biological, Salem, MA), 2.2 g/L sodium bicarbonate, pH to 7.4. A Bradford protein assay (Bio-Rad, Hercules, CA) was used to determine the protein concentration and membranes were diluted and stored at -80 °C for later use.

Radioligand competition binding experiments were conducted using thawed membranes on test day, each test compound was diluted into 10 half-log serial dilutions using 30% DMSO vehicle, starting from 1 mM or 100 μM concentration. To facilitate the solubilization of free-base compounds, 10 μL of glacial acetic acid was added along with the DMSO. Previously frozen membranes were diluted in fresh EBSS binding buffer to 200 μg/mL (for $hD_{2L}R$ or hD_3R) or 400 μg/mL (for hD_4R) for binding. Radioligand competition experiments were conducted in 96-well plates containing 300 μl fresh EBSS binding buffer, 50 μl of diluted test compound, 100 μl of membranes (20 μg/well total protein for $hD_{2L}R$ and hD_3R, and 50 μl of [^{3}H]*N*-methylspiperone radioligand diluted in binding buffer (0.4 nM final concentration; Perkin Elmer). Nonspecific

binding was determined using 10 µM (+)-butaclamol (Sigma-Aldrich, St. Louis, MO) and total binding was determined with 30% DMSO vehicle. All compound dilutions were tested in triplicate and the reaction incubated for 1 hour at RT. The reaction was terminated by filtration through Perkin Elmer Uni-Filter-96 GF/C plates, using a Brandel 96-Well Plates Harvester Manifold (Brandel Instruments, Gaithersburg, MD). The filters were washed (3 × 1 mL/well) of ice-cold binding buffer. Perkin Elmer MicroScint 20 Scintillation Cocktail (45 µL) was added to each well and filters were counted using a Perkin Elmer MicroBeta Microplate Counter. IC_{50} values for each compound were determined from dose-response curves and K_i values were calculated using the Cheng-Prusoff equation.[5] When a complete inhibition couldn't be achieved at the highest tested concentrations, K_i values have been extrapolated by constraining the bottom of the dose-response curves (= 0% residual specific binding) in the non-linear regression analysis. These analyses were performed using GraphPad Prism version 10.5 (GraphPad Software, San Diego, CA). All results were rounded to three significant figures. K_i values were determined from at least 3 independent experiments and are reported as means ± SEM.

Computational Modeling Studies:

Initial Model Preparation. The crystal structure used for Molecular Dynamics (MD) simulations was obtained from the Protein Data Bank (PDB ID: 5WIU)[6] with a resolution of 1.96 Å. The co-crystallized ligand, Nemonapride, was removed. Residues 1001–1106, corresponding to the thermostabilized apocytochrome b562RIL (BRIL) fusion partner, were deleted. To simplify the intracellular loop 3 (ICL3), it was truncated and replaced with a shorter segment consisting of residues 220–225 from the DRD4 sequence. Two disulfide bonds were introduced between cysteine pairs C108–C185 and C420–C423. Two aspartic acids at ASP80 and ASP132 were protonated to simulate the active conformation as per Zhang et al[7]. The resulting protein structure

was further refined using the Protein Preparation Wizard in Schrödinger Maestro[7b], which included assigning protonation states to ionizable residues and rebuilding the missing loop absent in the original PDB structure.

Membrane Preparation. The refined protein structure from the previous step was used for membrane embedding. To determine the correct membrane orientation, the structure was aligned using the Orientations of Proteins in Membranes (OPM) database[8]. This OPM-aligned model was then used as input for membrane packing with Packmol-memgen[9]. A lipid bilayer composed of a 9:1 molar ratio of phosphatidylcholine (POPC) to cholesterol (CHL1) was generated. Sodium and chloride ions were added to achieve a physiological salt concentration of 150 mM NaCl. The system was enclosed in a rectangular box with a 10 Å buffer in the x, y, and z dimensions. To solvate the system, 15 Å water layers were added above and below the membrane. The final membrane-protein complex was used as input for tleap in AMBER[10, 11] to complete system preparation for MD simulations.

Apo System Preparation. System preparation was completed using tleap. The AMBER FF19SB[12] force field was applied to the protein, Lipid21[13] to the membrane lipids, and the OPC[14] water model for solvation. To enable a larger integration timestep of 4 femtoseconds (fs), hydrogen mass repartitioning[15] (HMR) was performed using ParmEd[16].

Molecular Dynamics (MD) Protocol for Apo-D$_4$R. All MD simulations were conducted using the AMBER suite. The system underwent five sequential energy minimization steps. First, the model was subjected to 5,000 cycles of steepest descent followed by 5,000 cycles of conjugate gradient minimization with a restraint weight of 25 kcal/(mol·Å^2) applied to both the membrane and protein. In the second step, the same minimization procedure was repeated with the restraint

weight reduced to 5 kcal/(mol·Å^2) on the membrane and protein. The third step applied the same protocol with restraints of 5 kcal/(mol·Å^2) on the protein only. In the fourth step, restraints on the protein were reduced to 1 kcal/(mol·Å^2). Finally, the system was minimized with 5,000 cycles of steepest descent and 10,000 cycles of conjugate gradient without any restraints.

The system was then gradually heated from 0 to 100 K over 5 picoseconds (ps), followed by heating to 310 K over 100 ps while maintaining the previous restraints. Equilibration was performed using the Berendsen barostat with a target pressure of 1.0 atm. Initially, restraints of 5 kcal/(mol·Å^2) were applied to both the ligand and protein backbone for 2 nanoseconds (ns). This was followed by an additional 2 ns with the same restraints applied to the ligand and α-carbons of the protein. In the final equilibration phase, all atoms were allowed to move freely for 100 ns.

The production MD simulation was run for 2.5 microseconds (µs) using the Monte Carlo barostat with a target pressure of 1.0 atm. Throughout all stages, the SHAKE algorithm was used to constrain bonds involving hydrogen atoms, and hydrogen mass repartitioning (HMR) was applied to enable a 4 femtosecond (fs) integration timestep. A 10 Å nonbonded cutoff was used, and long-range electrostatics were treated with the particle mesh Ewald method. Temperature was maintained at 310 K using a Langevin thermostat[17] with a collision frequency of 2.0 ps^{-1}. Pressure control was achieved with a relaxation time of 2.0 ps. For compound docking studies, a representative frame from the first 100 ns of simulation was extracted in which the extended binding pocket (EBP) was clearly visible.

Ligand Preparation and Docking. Compounds **1** through **9** were initially sketched in 2D using Schrödinger Maestro's 2D Sketcher tool and subsequently converted to 3D structures. Conformers were generated using the LigPrep module within a pH range of 7.0 ± 2.0. Only compounds

containing a quaternary amine were retained for docking studies, yielding one conformer per compound.

Docking was performed using Schrödinger's Glide[18-21] in Standard Precision (SP) mode. A receptor grid was generated centered on the conserved D115^3.32 residue, with a box size of 20 × 20 × 20 Å. Each ligand was docked with the SP protocol, generating up to 20 poses per compound, followed by post-docking minimization. A single pose was selected for each compound if it satisfied the following criteria: (1) formation of a hydrogen bond between the ligand's quaternary amine and D115, and (2) proper orientation of the modified ring within the extended binding pocket (EBP).

Selected ligand poses were subjected to geometry optimization at the HF/6-31G* level of theory using Gaussian16. Electrostatic potential (ESP) charges were calculated at the same level, and restrained electrostatic potential (RESP) charges were derived using the RESP method as implemented in the Antechamber module of AmberTools. Ligand topology and coordinate files were generated using tleap.

Ligand-Bound Model Preparation Protocol. For compounds **1–9**, system preparation followed the same procedures as the apo model, including model refinement, membrane embedding with Packmol-Memgen, and system setup with tleap. The key difference was the inclusion of each ligand, which required the use of RESP charges as described in the Ligand Preparation and Docking section. Ligands were parameterized using the GAFF2[22] force field within tleap to generate the final ligand-bound models for molecular dynamics simulations.

Molecular Dynamics (MD) Protocol for Ligand-Bound D_4R. MD simulations were performed using the AMBER suite. The system underwent two energy minimization steps. In the first, 500

cycles of steepest descent were followed by 4,500 cycles of conjugate gradient minimization using pmemd. The second minimization applied 5,000 cycles of steepest descent followed by 5,000 cycles of conjugate gradient using pmemd.cuda[23, 24]. Both steps were conducted with a nonbonded cutoff of 10.0 Å and without applying the SHAKE algorithm.

The system was then gradually heated in two phases. First, it was heated from 0 K to 100 K over 5 picoseconds (ps) with weak positional restraints of 5 kcal/(mol·Å²) applied to all atoms except water and ions. This was followed by heating from 100 K to 303 K over 500 ps under the same restraints. During the second heating phase, the Berendsen barostat was enabled to simultaneously ramp temperature and pressure, targeting 1.0 atm with anisotropic pressure scaling and a relaxation time of 2.0 ps.

Subsequent equilibration involved gradual release of positional restraints on the protein. In the fifth step, the system was simulated for 1 nanosecond (ns) with restraints of 5 kcal/(mol·Å²) applied to the ligand and protein backbone heavy atoms (Cα, C, and N). Semi-isotropic pressure scaling was used, maintaining constant surface tension in the xy-plane with a value of 0 dyne/cm. In the sixth step, the system underwent an additional 1 ns with restraints maintained on the ligand and protein Cα atoms. Finally, a 50 ns unrestrained equilibration phase was followed by a 50 ns production run. Pressure control parameters were kept constant during these stages.

For all non-minimization phases, the SHAKE algorithm was applied to constrain bonds involving hydrogen atoms. Hydrogen mass repartitioning (HMR) enabled the use of a 4 femtosecond (fs) timestep. A nonbonded cutoff of 10 Å was applied, and long-range electrostatics were treated using the particle mesh Ewald (PME) method. Temperature was controlled using a Langevin thermostat

with a collision frequency of 1.0 ps^{-1}. Pressure was maintained at 1.0 atm throughout all barostat-controlled steps using a relaxation time of 1.0 ps.

Post-MD Analyses. All molecular dynamics trajectory analyses were performed using Cpptraj[25]. Structural visualization and model images were generated with ChimeraX v1.8[26]. Post-MD binding energy analyses were carried out using both DeepAtom[27] and Schrödinger Maestro.

To assess system stability, root mean square deviation (RMSD) values were calculated for both the ligand and the protein backbone. Clustering of the final 50 nanoseconds of the simulation was performed using the k-means[28] algorithm to identify representative conformations of the ligand-bound complexes for subsequent analysis.

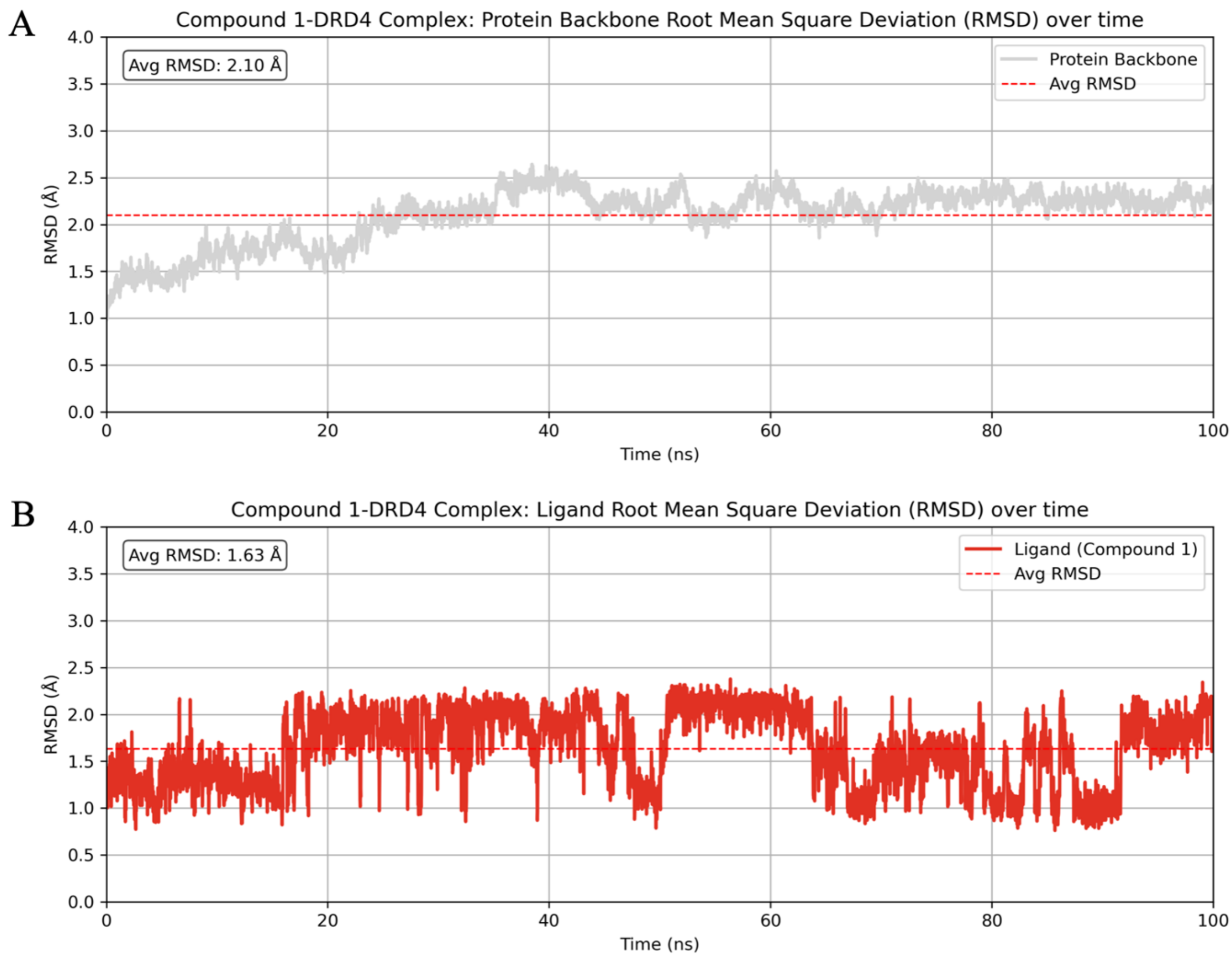

Figure S1. RMSD values from the compound **1**-DRD4 MD simulation. **(A)** RMSD of the protein backbone atoms (alpha carbon, carbon, and nitrogen). **(B)** RMSD of the ligand atoms.

A

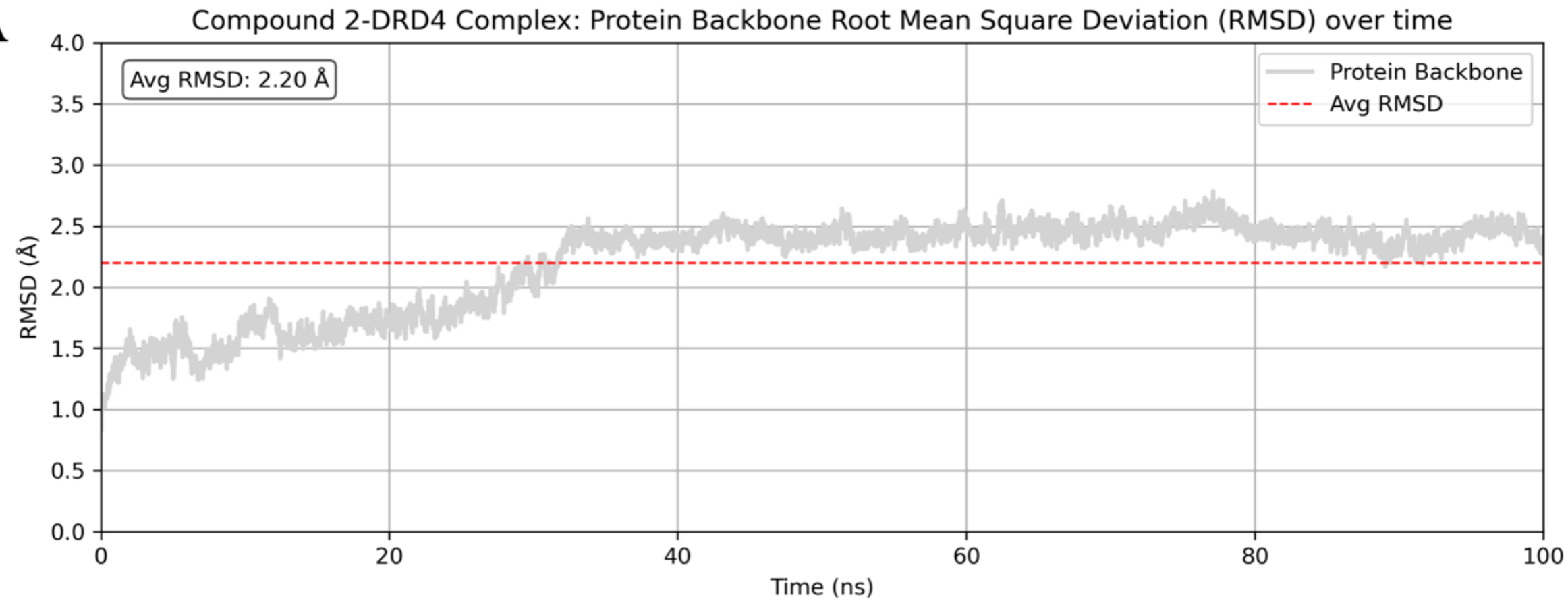

B

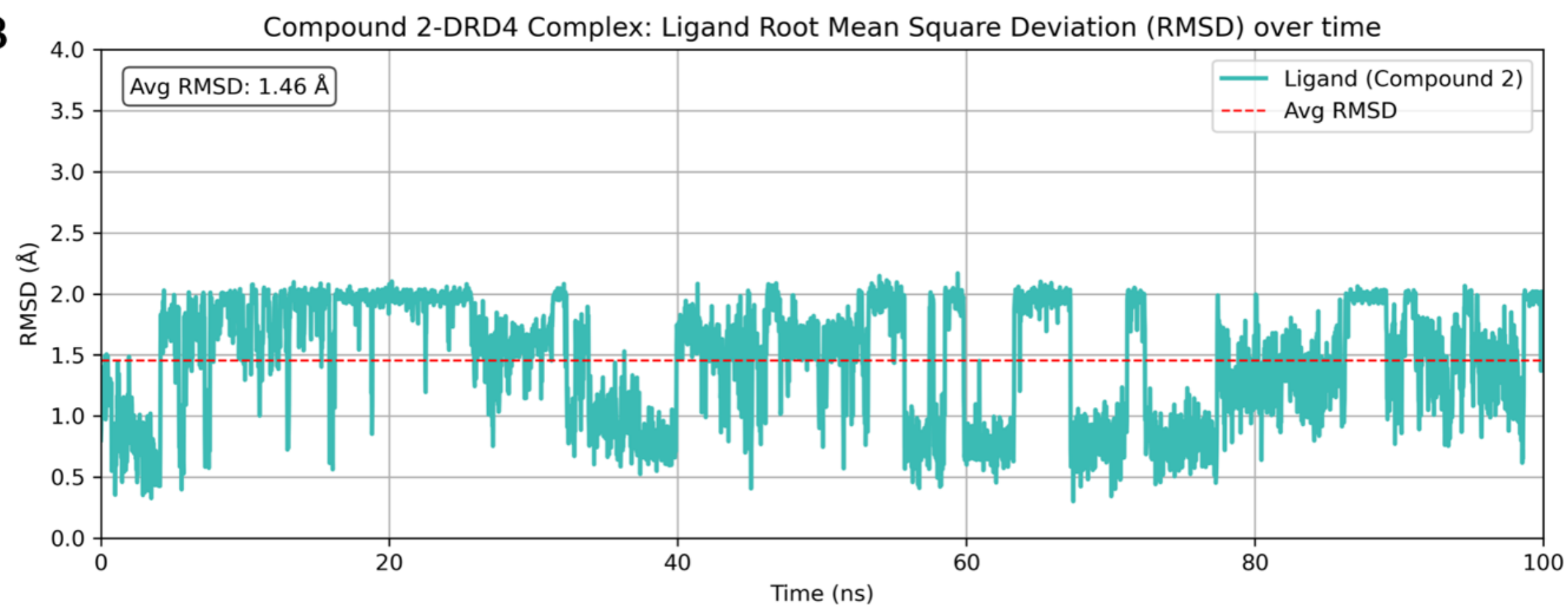

Figure S2. RMSD values from the compound **2**-DRD4 MD simulation. **(A)** RMSD of the protein backbone atoms (alpha carbon, carbon, and nitrogen). **(B)** RMSD of the ligand atoms.

A
B

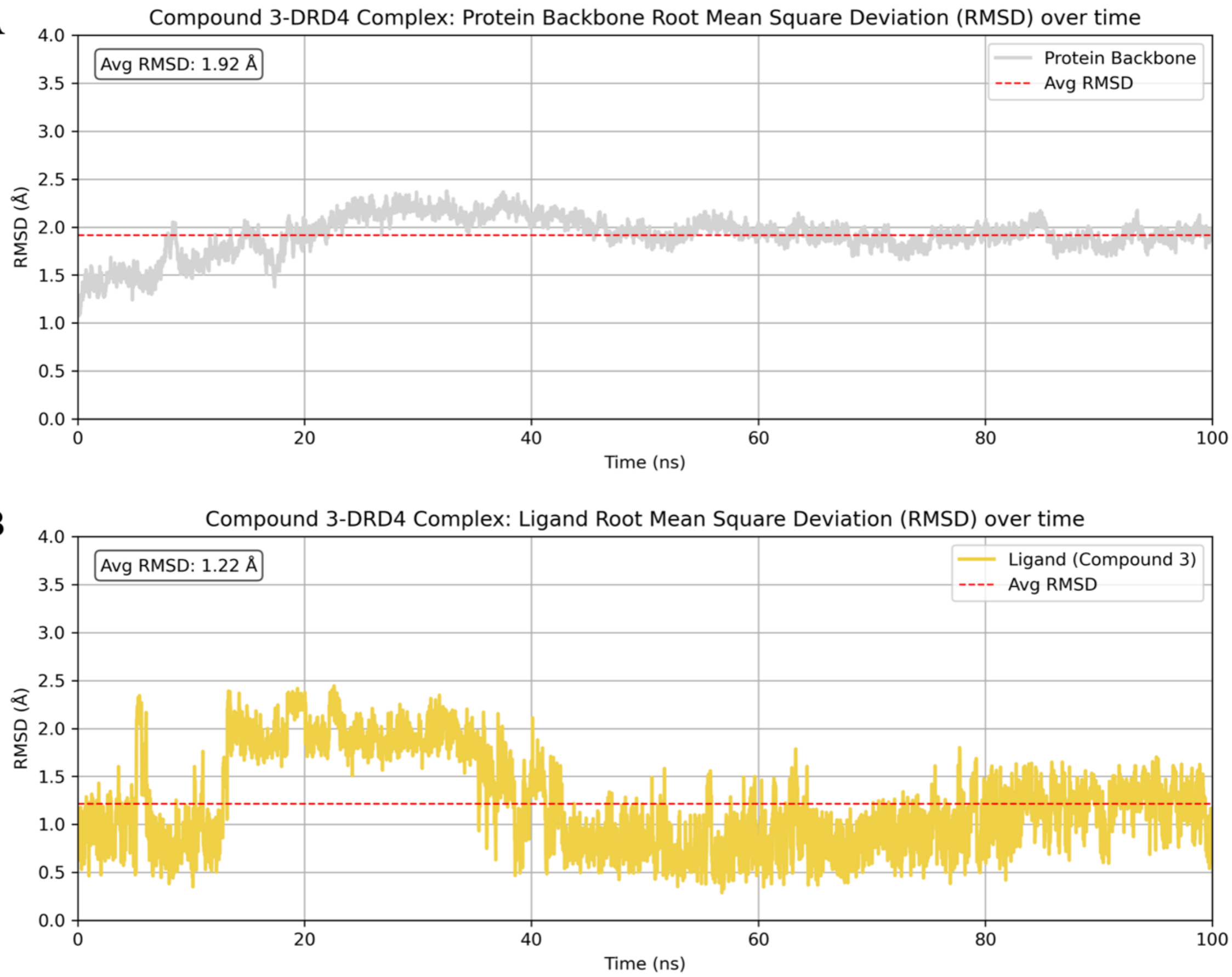

Figure S3. RMSD values from the compound **3**-DRD4 MD simulation. **(A)** RMSD of the protein backbone atoms (alpha carbon, carbon, and nitrogen). **(B)** RMSD of the ligand atoms.

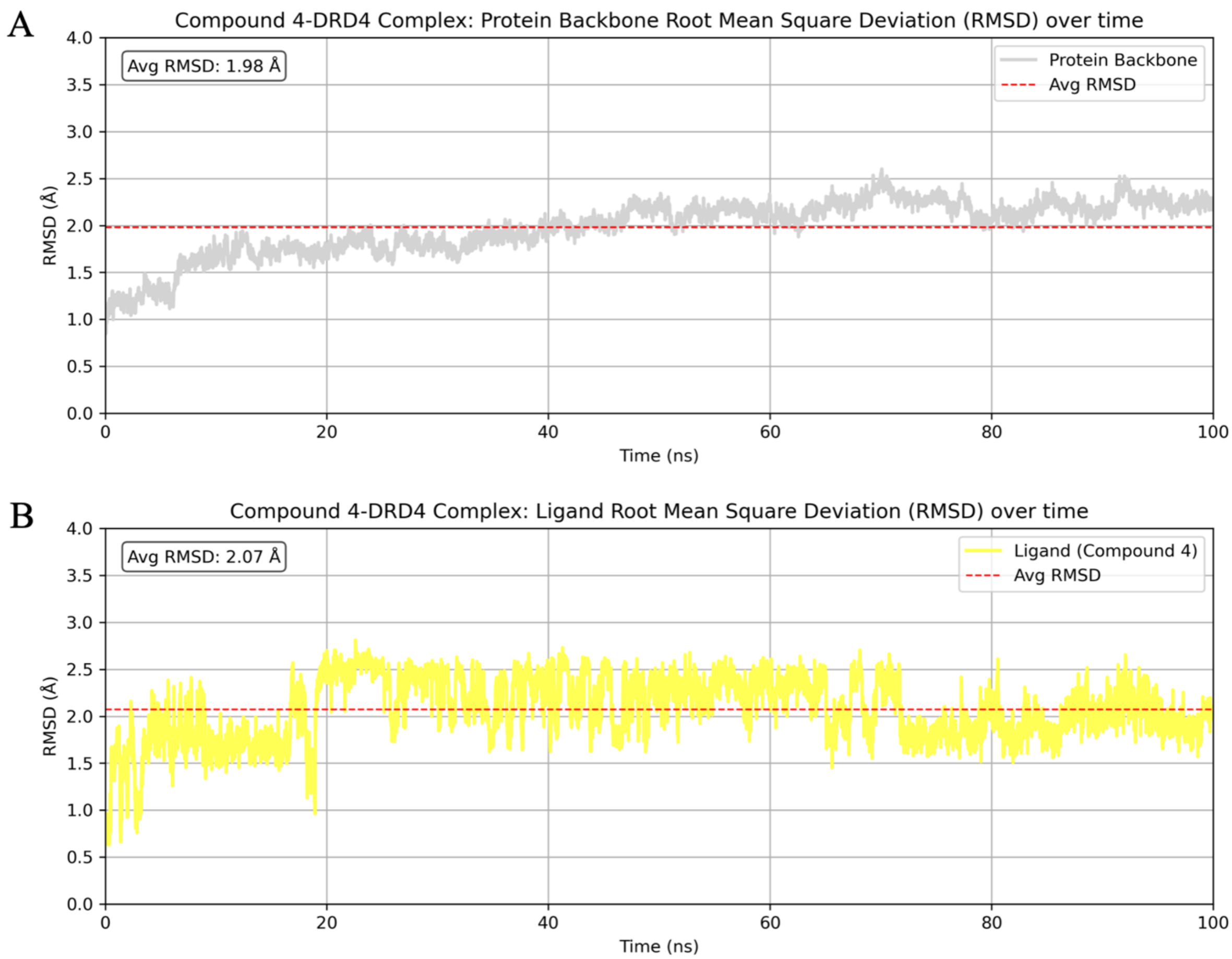

Figure S4. RMSD values from the compound **4**-DRD4 MD simulation. **(A)** RMSD of the protein backbone atoms (alpha carbon, carbon, and nitrogen). **(B)** RMSD of the ligand atoms.

A

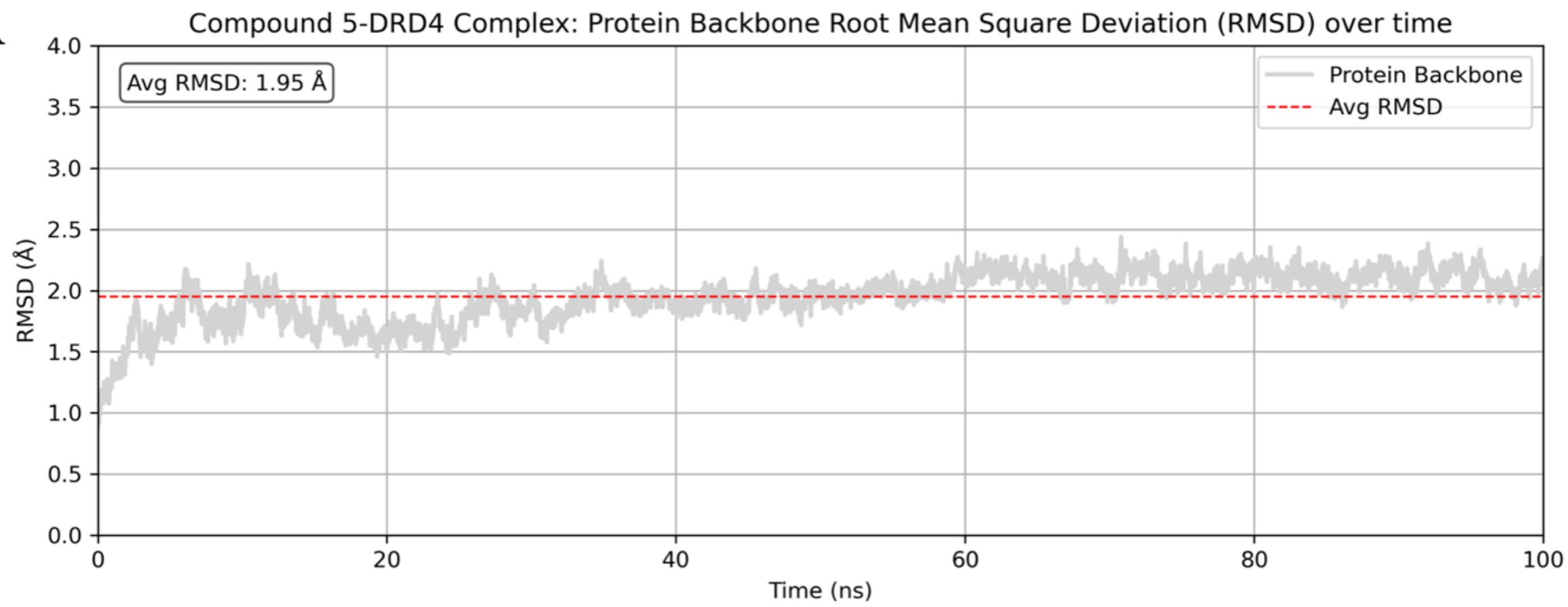

B

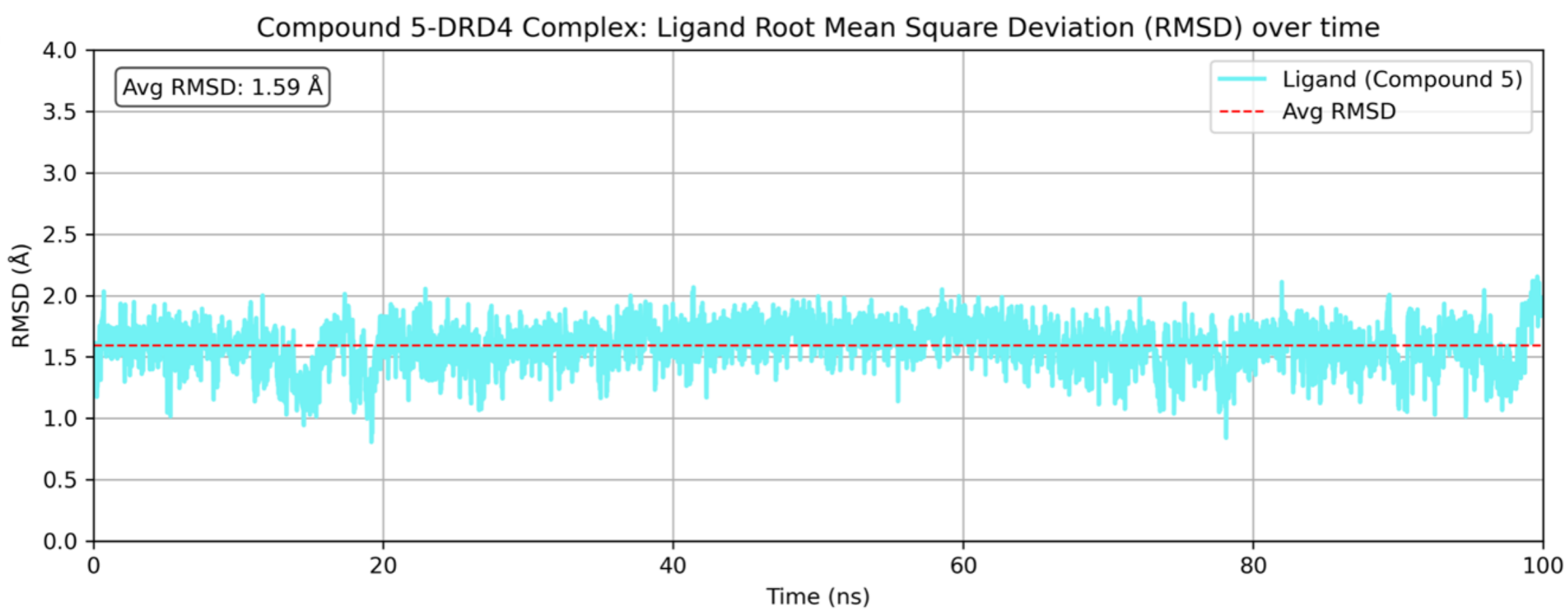

Figure S5. RMSD values from the compound **5**-DRD4 MD simulation. **(A)** RMSD of the protein backbone atoms (alpha carbon, carbon, and nitrogen). **(B)** RMSD of the ligand atoms.

A

B

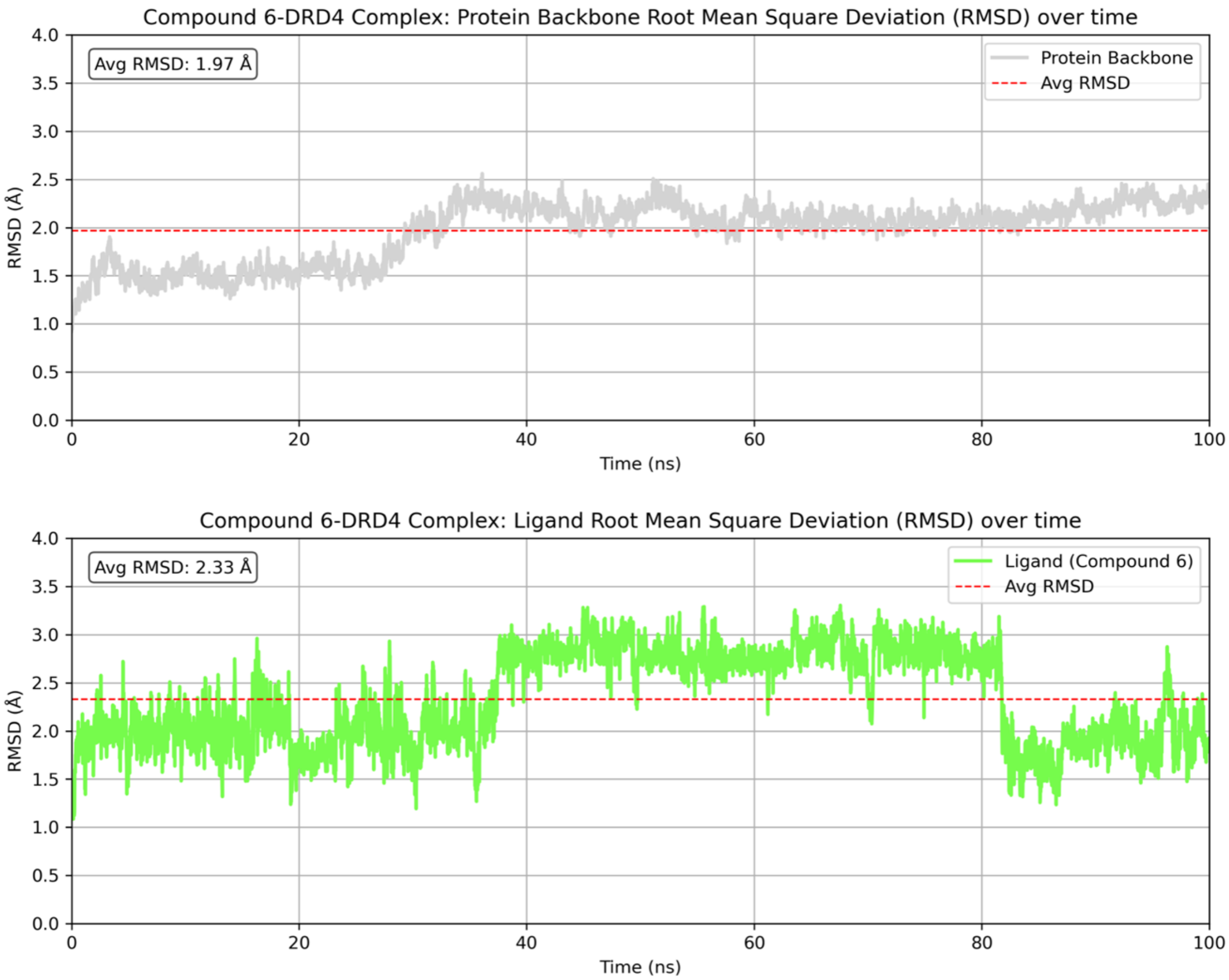

Figure S6. RMSD values from the compound **6**-DRD4 MD simulation. **(A)** RMSD of the protein backbone atoms (alpha carbon, carbon, and nitrogen). **(B)** RMSD of the ligand atoms.

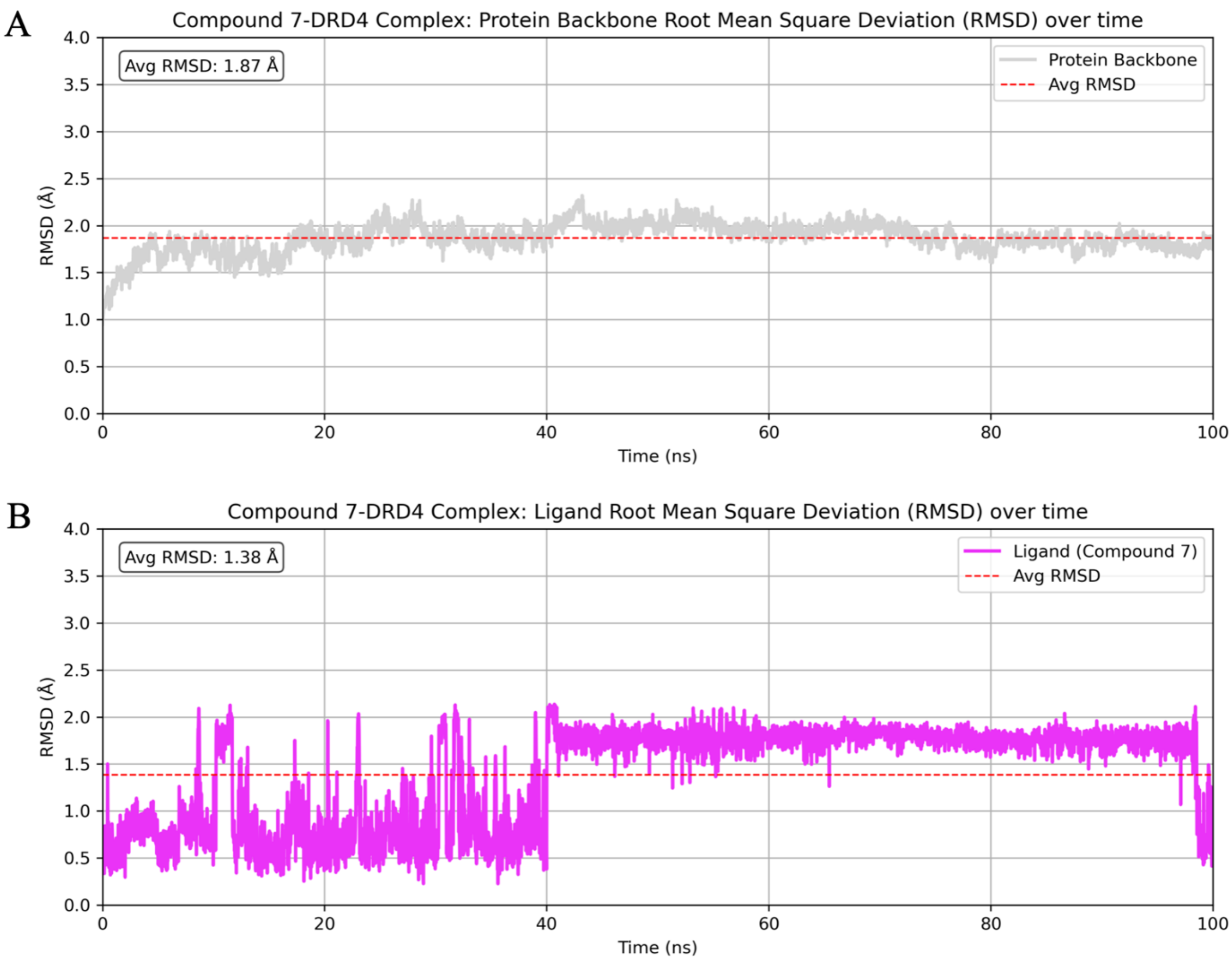

Figure S7. RMSD values from the compound **7**-DRD4 MD simulation. **(A)** RMSD of the protein backbone atoms (alpha carbon, carbon, and nitrogen). **(B)** RMSD of the ligand atoms.

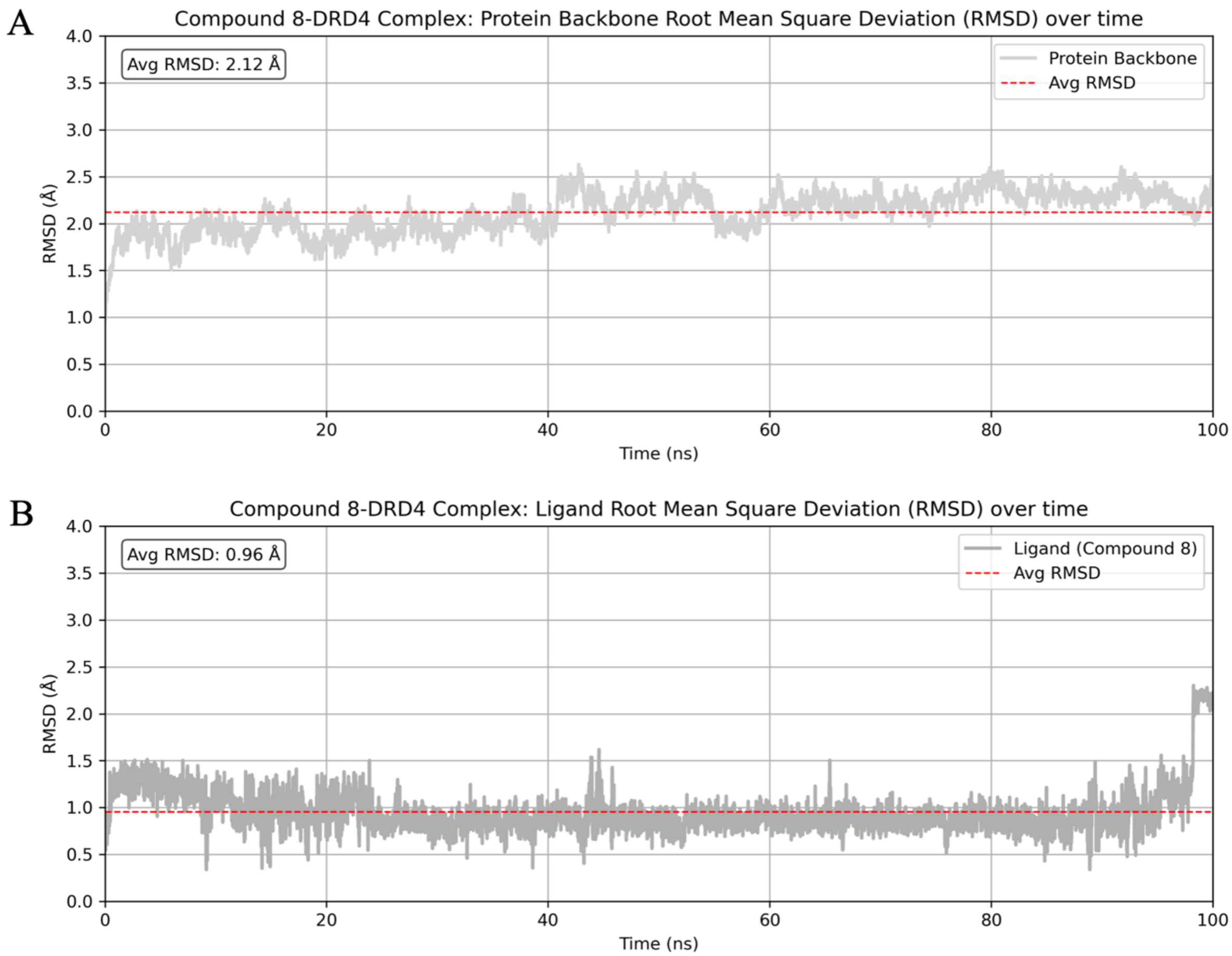

Figure S8. RMSD values from the compound **8**-DRD4 MD simulation. **(A)** RMSD of the protein backbone atoms (alpha carbon, carbon, and nitrogen). **(B)** RMSD of the ligand atoms.

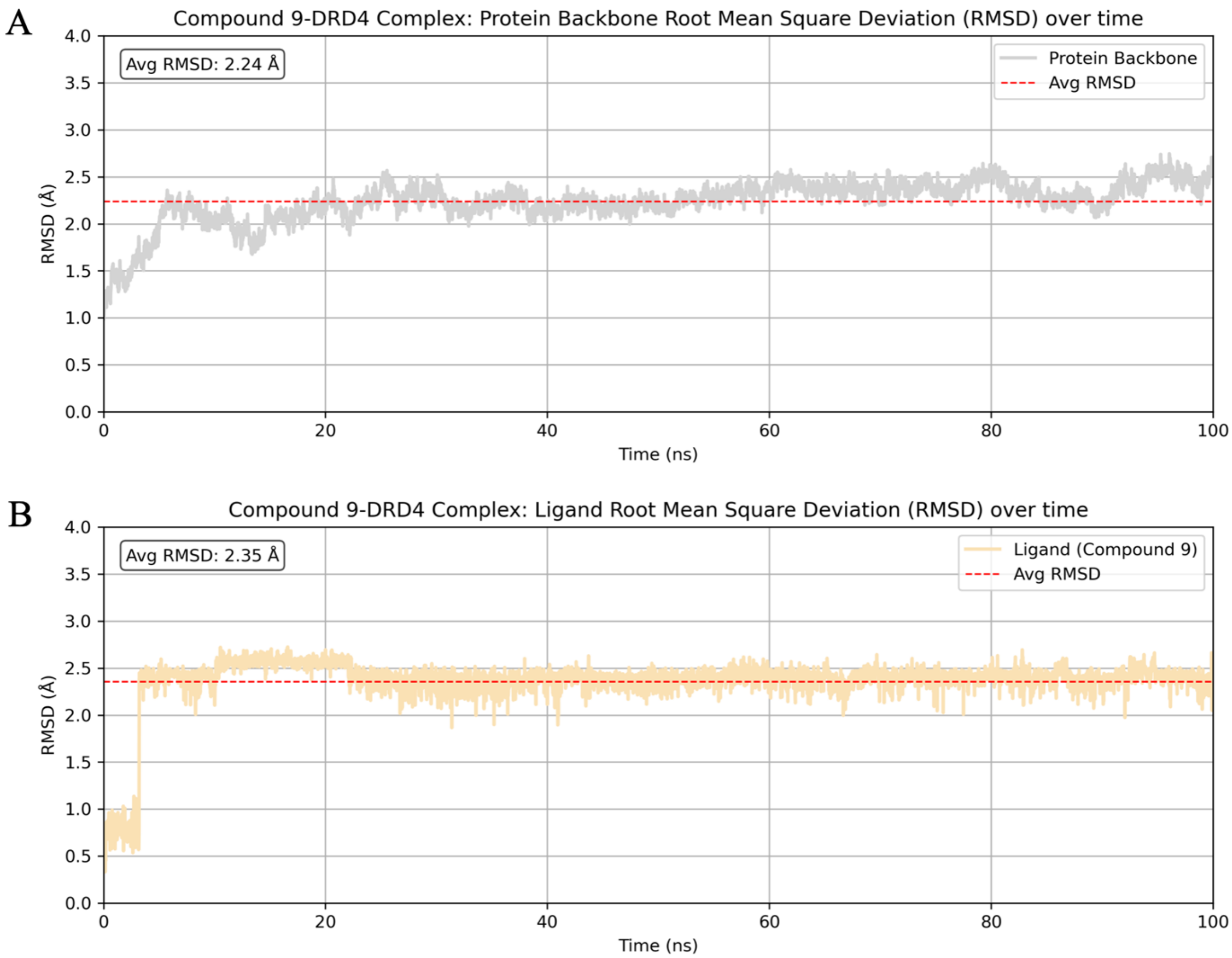

Figure S9. RMSD values from the compound **9**-DRD4 MD simulation. **(A)** RMSD of the protein backbone atoms (alpha carbon, carbon, and nitrogen). **(B)** RMSD of the ligand atoms.

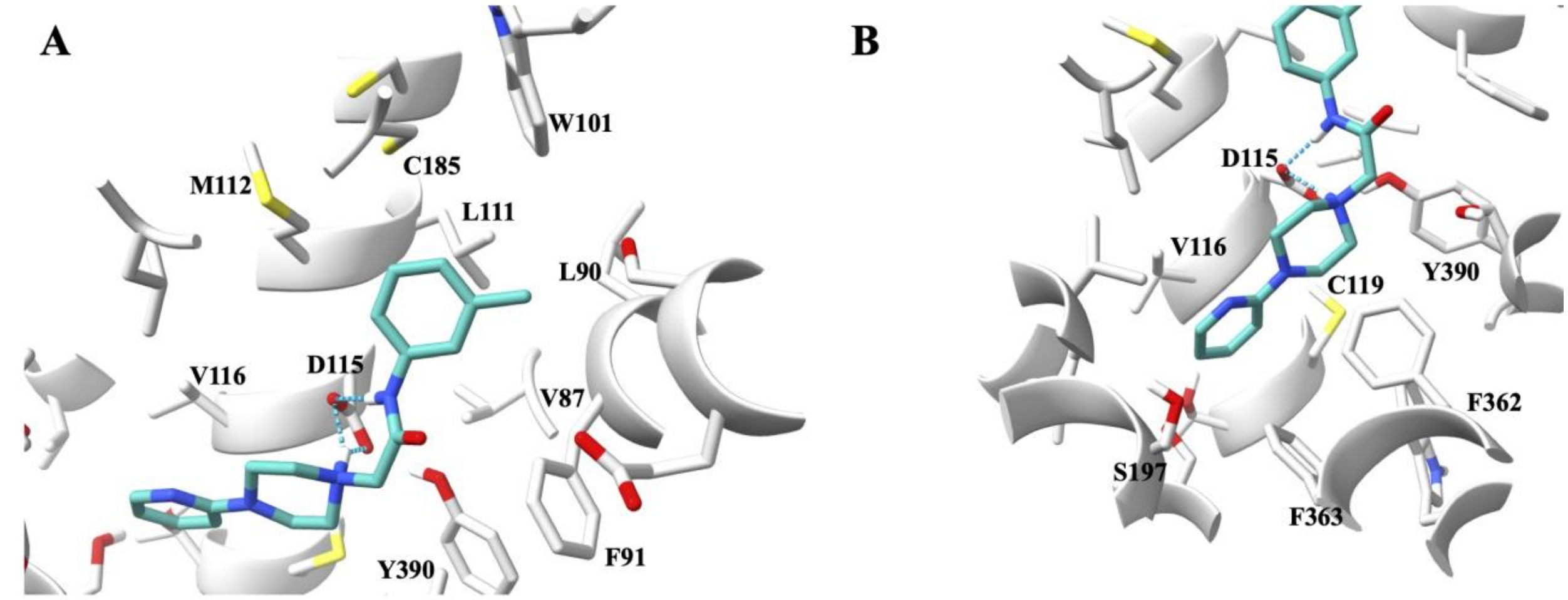

Figure S10. Image of the compound **2**-DRD4 Complex. **(A)** View of residues in the EBP that interact with compound **2**. **(B)** View of residues in the OBP that interact with compound **2**.

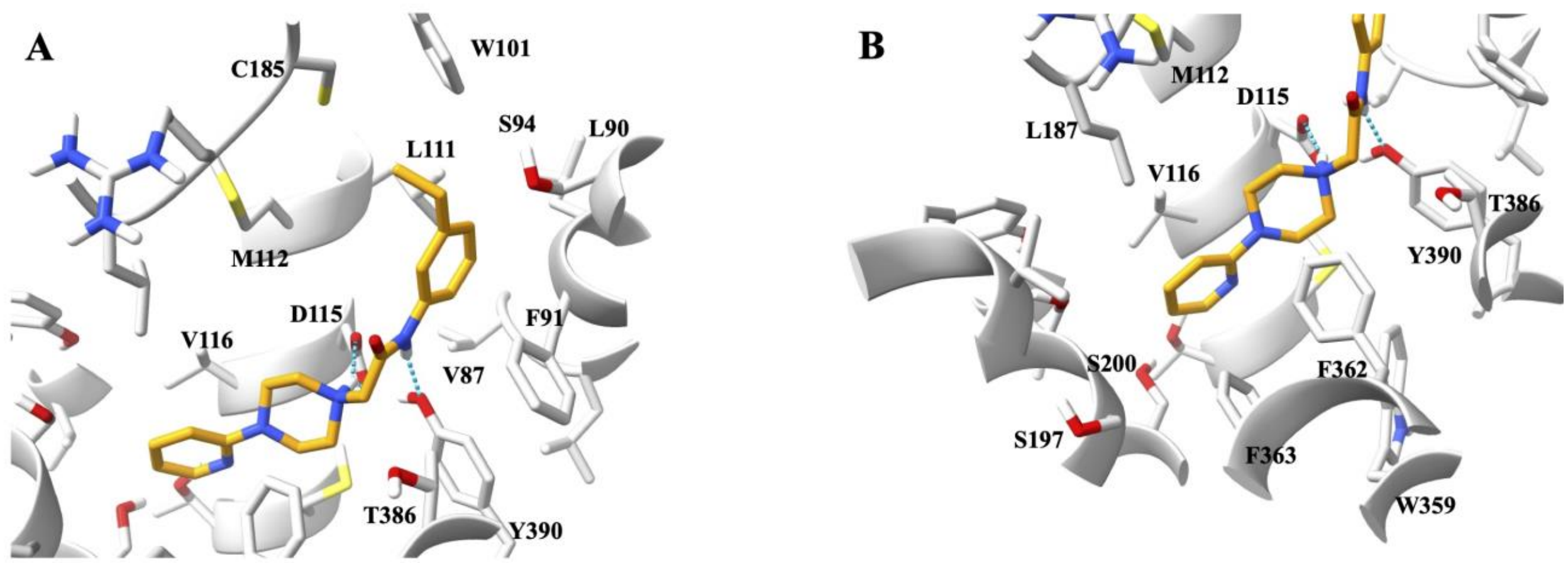

Figure S11. Image of the compound **3**-DRD4 Complex. **(A)** View of residues in the EBP that interact with compound **3**. **(B)** View of residues in the OBP that interact with compound **3**.

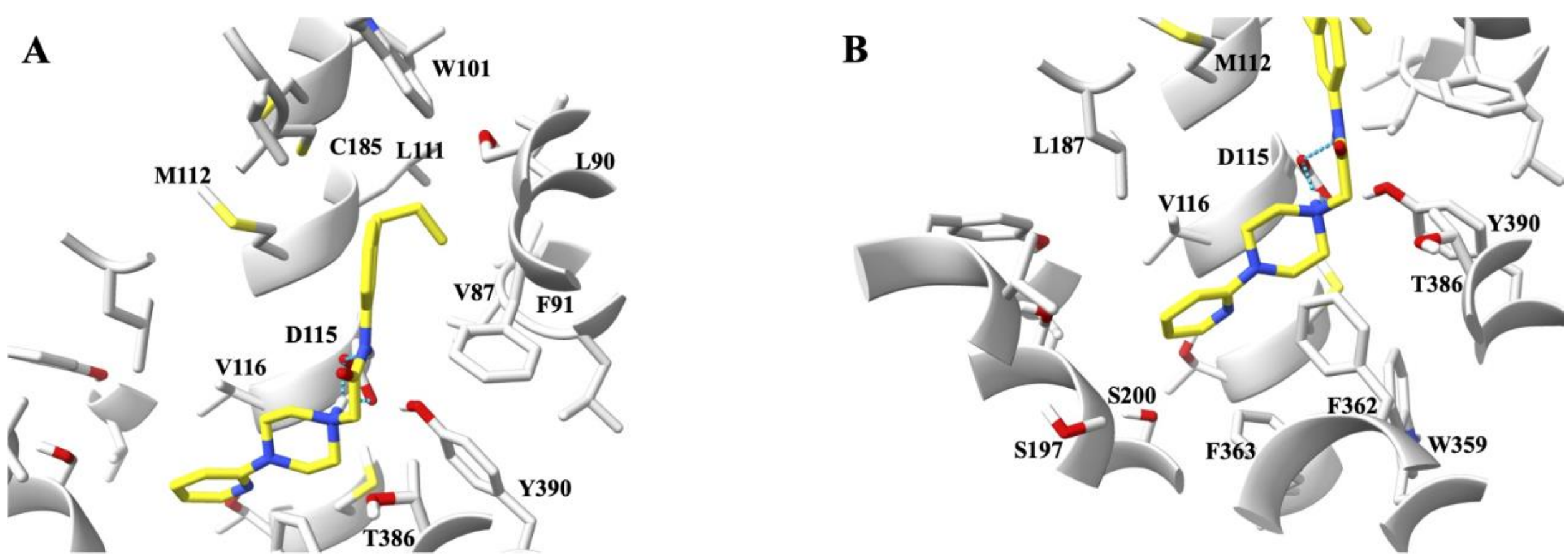

Figure S12. Image of the compound **4**-DRD4 Complex. **(A)** View of residues in the EBP that interact with compound **4**. **(B)** View of residues in the OBP that interact with compound **4**.

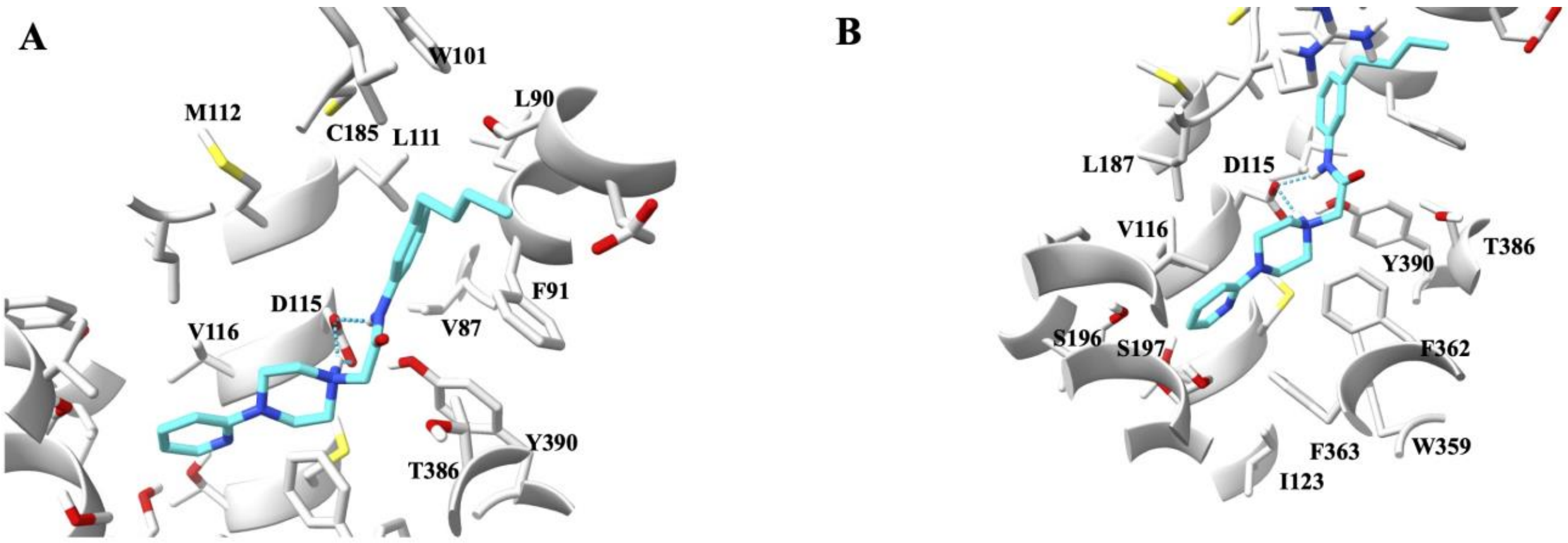

Figure S13. Image of the compound **5**-DRD4 Complex. **(A)** View of residues in the EBP that interact with compound **5**. **(B)** View of residues in the OBP that interact with compound **5**.

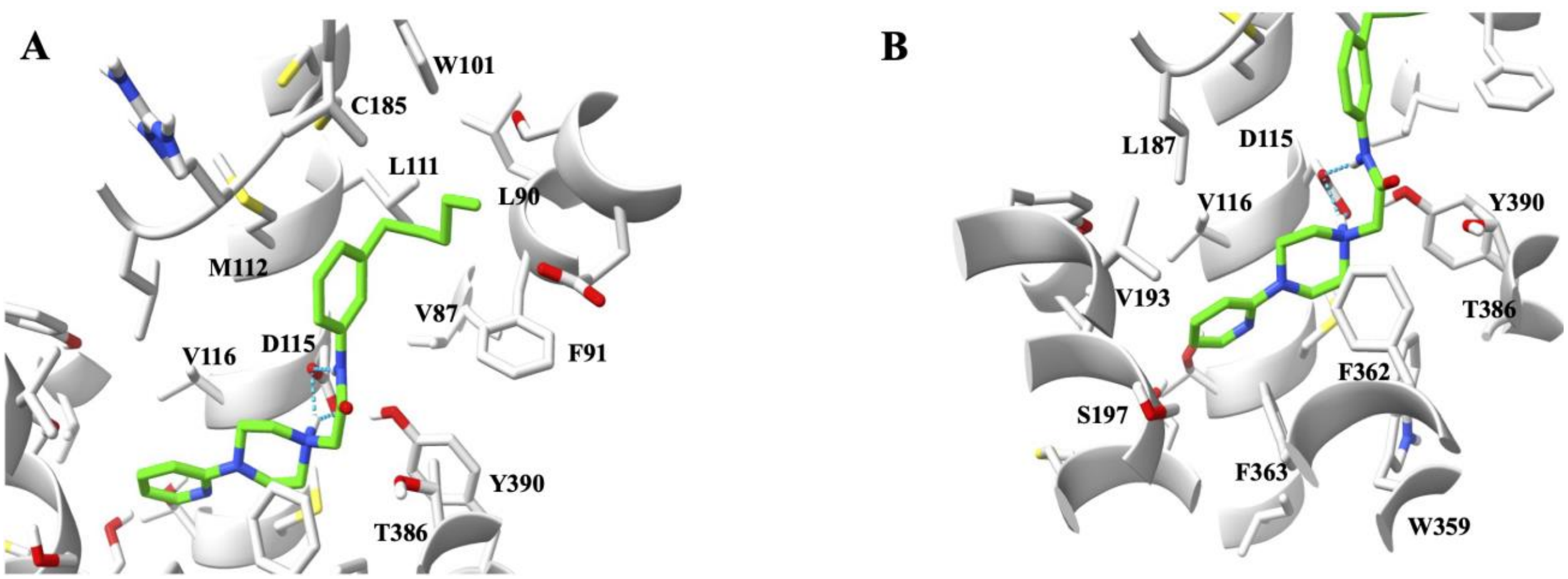

Figure S14. Image of the compound **6**-DRD4 Complex. **(A)** View of residues in the EBP that interact with compound **6**. **(B)** View of residues in the OBP that interact with compound **6**.

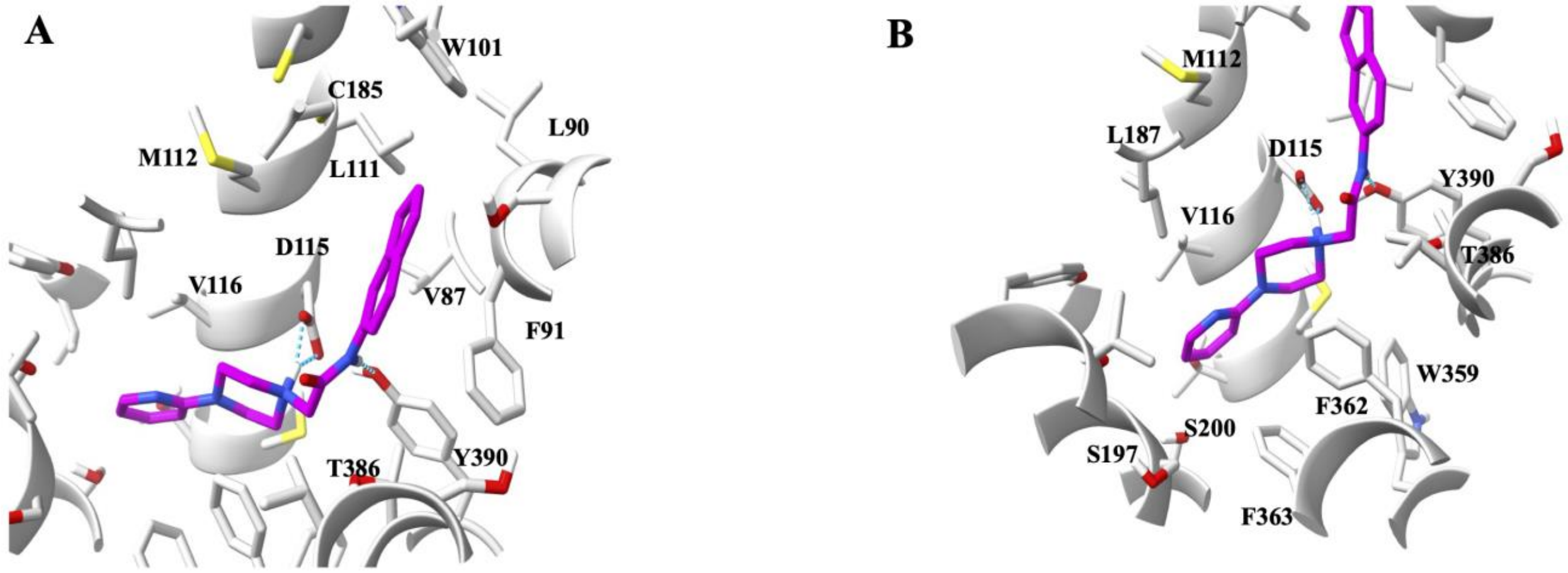

Figure S15. Image of the compound **7**-DRD4 Complex. **(A)** View of residues in the EBP that interact with compound **7**. **(B)** View of residues in the OBP that interact with compound **7**.

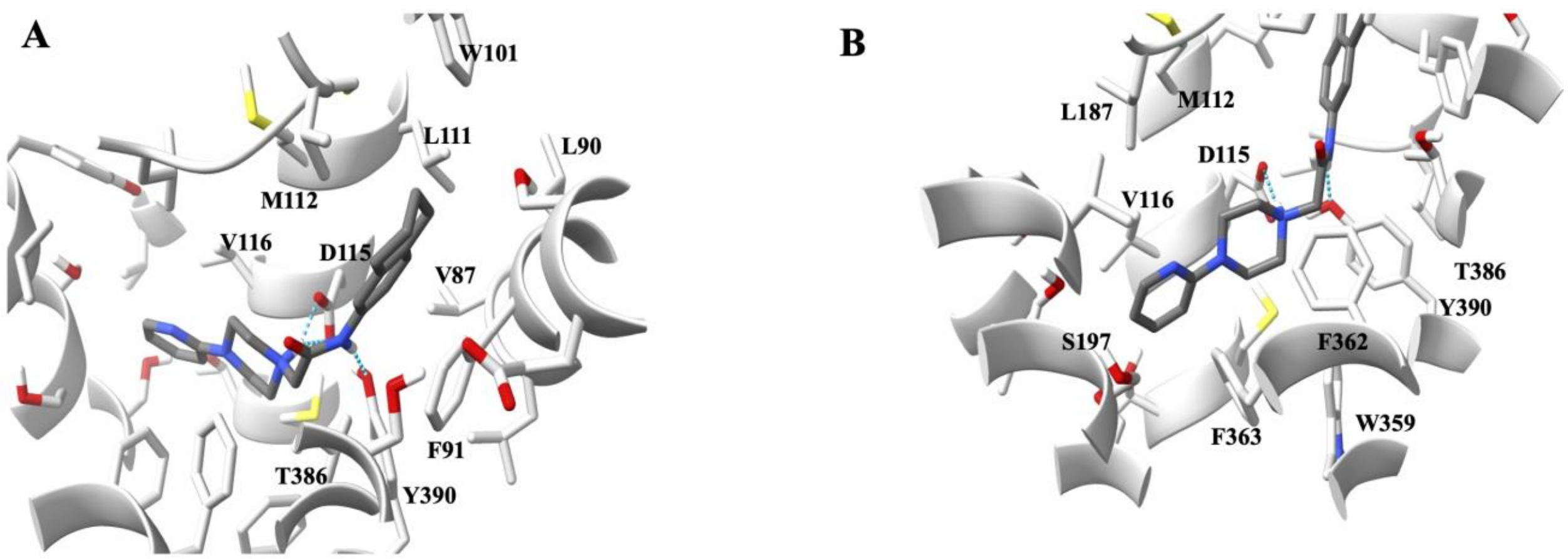

Figure S16. Image of the compound **8**-DRD4 Complex. **(A)** View of residues in the EBP that interact with compound **8**. **(B)** View of residues in the OBP that interact with compound **8**.

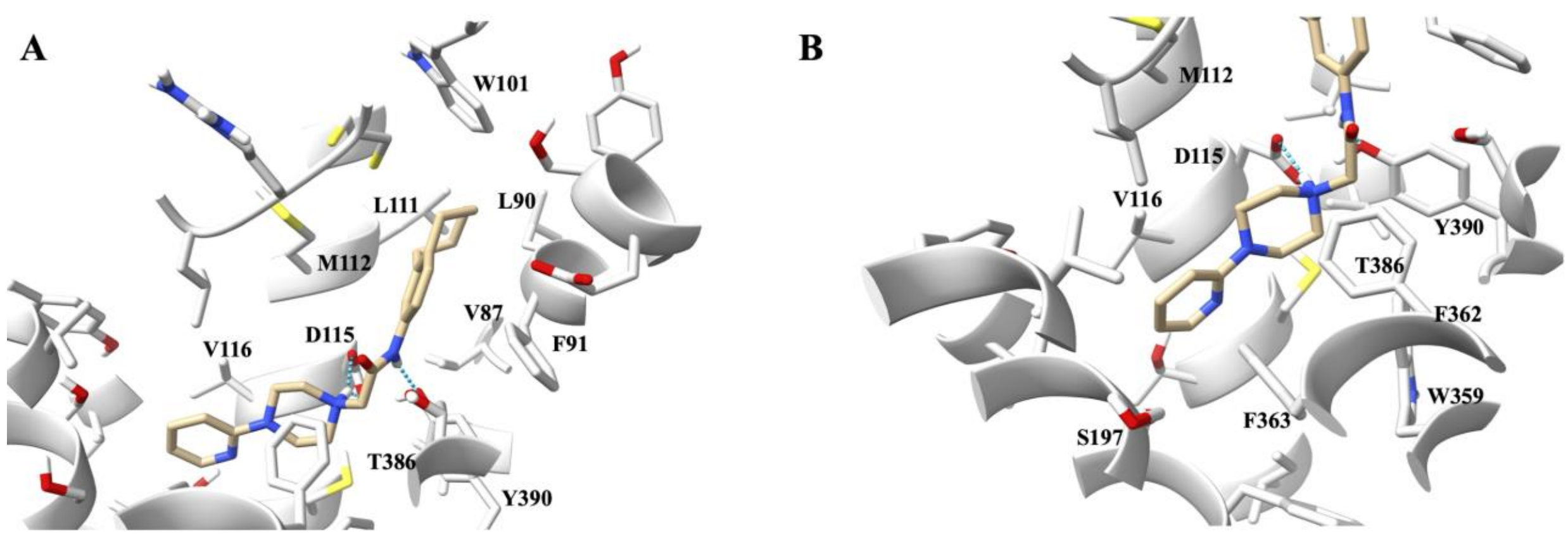

Figure S17. Image of the compound **9**-DRD4 Complex. **(A)** View of residues in the EBP that interact with compound **9**. **(B)** View of residues in the OBP that interact with compound **9**.

Table S1. Physicochemical Properties of the final Compounds.

No.	cLogP [a]	MW [a]	TPSA [a]	pKa [b]	HBD [b]	cLogD [c]	Smiles
**1**	2.51	309.41	44.7	7.2	1	2.60	O=C(CN1CCC(C2=NC=CC=C2)CC1)NC3=CC(C)=CC=C3
**2**	2.46	310.40	47.94	8.5	1	2.66	O=C(CN1CCN(C2=NC=CC=C2)CC1)NC3=CC(C)=CC=C3
**3**	2.87	324.43	47.94	8.5	1	3.10	O=C(CN1CCN(C2=NC=CC=C2)CC1)NC3=CC(CC)=CC=C3
**4**	3.29	338.46	47.94	8.5	1	3.55	O=C(CN1CCN(C2=NC=CC=C2)CC1)NC3=CC(CCC)=CC=C3
**5**	3.71	352.48	47.94	8.5	1	3.99	O=C(CN1CCN(C2=NC=CC=C2)CC1)NC3=CC(CCCC)=CC=C3
**6**	4.12	366.51	47.94	8.5	1	4.44	O=C(CN1CCN(C2=NC=CC=C2)CC1)NC3=CC(CCCCC)=CC=C3
**7**	2.86	336.44	47.94	8.5	1	3.15	O=C(CN1CCN(C2=NC=CC=C2)CC1)NC3=CC4=C(CCC4)C=C3
**8**	3.28	350.47	47.94	8.5	1	3.59	O=C(CN1CCN(C2=NC=CC=C2)CC1)NC3=CC4=C(CCCC4)C=C3
**9**	3.70	364.49	47.94	8.5	1	4.09	O=C(CN1CCN(C2=NC=CC=C2)CC1)NC3=CC4=C(CCCCC4)C=C3

[a] Calculated logP (cLogP), molecular weight (MW), and topological polar surface area (TPSA) were obtained using ChemDraw, version 23.1.1. [b] The ionization constant for the most basic pKa center and the number of hydrogen bond donors (HBDs) were determined via the Xundrug website,[1] using the compound's SMILES exported from ChemDraw. [c] Calculated logD (cLogD) value was estimated at pH 7.4 using the ChemAxon Marvin version.[1]

Table S2. CNS Multiparameter Optimization (MPO) Score of compound **LAB-01-26** using Pfizer's Active CNS MPO Calculator.

CNS MPO Calculator		
**Property**	**Value**	**T0**
ClogP	3.71	0.65
ClogD	3.99	0.00
TPSA	47.94	1.00
MW	352.48	1.00
HBD	1	0.83
pKa	8.5	0.75
	**CNS MPO**	**4.2**

To calculate the CNS MPO score, using **LAB-01-26** as an example, the values of these parameters of compound **LAB-01-26** were entered into an active CNS MPO calculator provided by Pfizer, as shown in **Table S2**, and the CNS MPO score was subsequently determined.[1] The CNS MPO scores of all compounds are summarized in **Table S**.

Table S3. CNS Multiparameter Optimization (MPO) Scores for All Compounds.

**Compound Code**	**No.**	**CNS MPO score**
**A-412997**	**1**	5.5
**CAB-02-140**	**2**	5.3
**CAB-02-017**	**3**	5.0
**LAB-01-023**	**4**	4.7
**LAB-01-026**	**5**	4.2
**LAB-01-029**	**6**	4.0
**TL-I-59**	**7**	5.0
**TL-I-60**	**8**	4.6
**TL-I-70**	**9**	4.2

CNS MPO value and method of calculation. Six physicochemical parameters must be evaluated for each compound, including the calculated logP water/octanol partitioning prediction (cLogP); molecular weight (MW); topological polar surface area (TPSA); ionization constant for the most pKa center (pKa), number of hydrogen bond donors (HBD), and calculated predicted water/octanol partition value at pH 7.4 (cLogD).[1] The physicochemical properties of all compounds are summarized in **Table S1**.

Table S4. Microanalysis data.

Compound	No	C	H	N	C	H	N
		Calculated			Found		
**LAB-01-023**	4	55.95	7.04	13.05	55.73	7.03	12.78
**LAB-01-026**	5	56.31	7.31	12.51	56.31	7.28	12.34
**LAB-01-029**	6	57.77	7.49	12.25	57.60	7.55	12.08
**TL-I-59**	7	56.21	6.6	13.11	56.09	6.22	12.72
**TL-I-60**	8	56.21	6.85	12.69	57.42	6.68	12.51
***TL-I-70**	9						

*Compound purity was determined by using HPLC and it's greater than 95 %. See Figure S18.

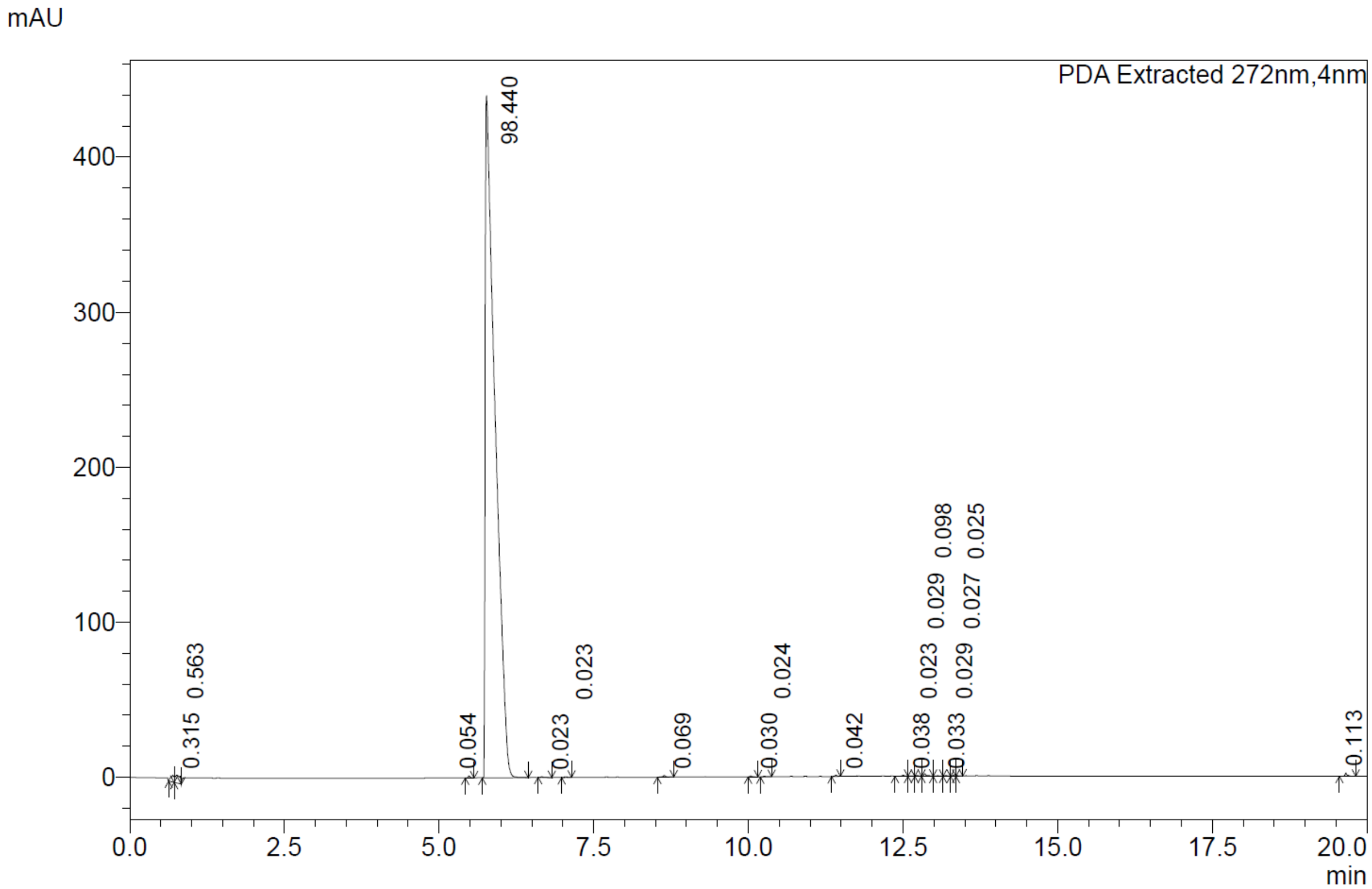

Figure S18. HPLC traces of compound **9**.

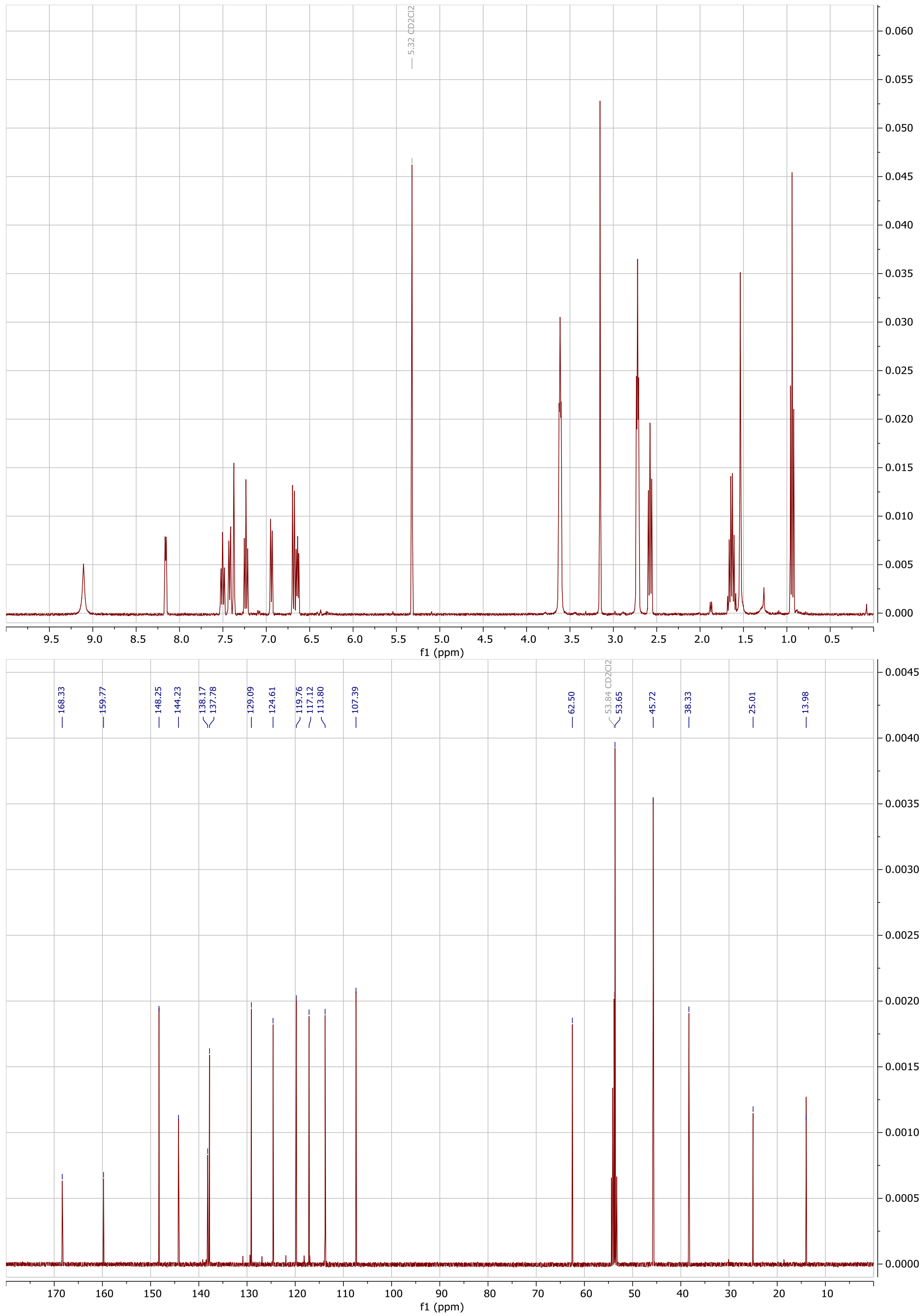

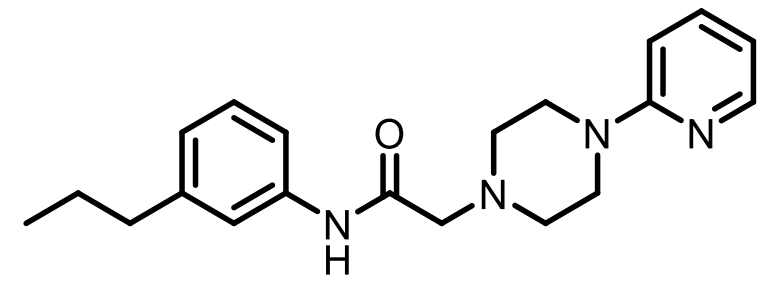

Figure S19. ^{1}H and ^{13}C NMR spectrum for compound 4

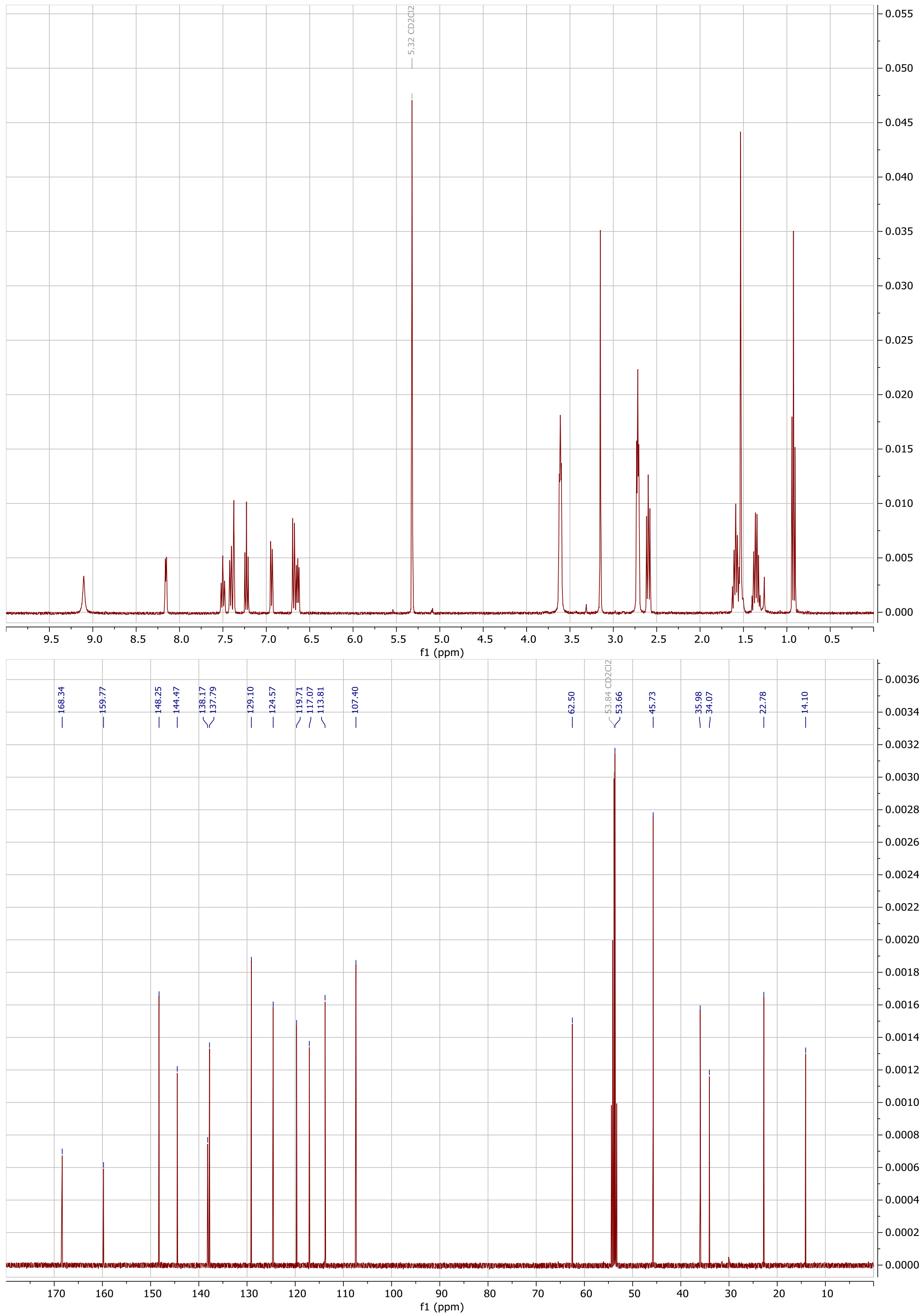

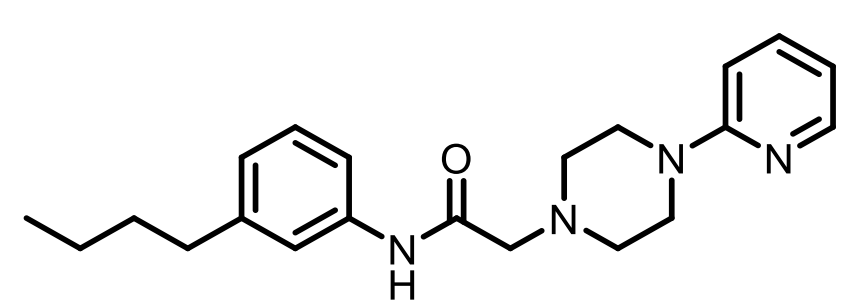

Figure S20. ^{1}H and ^{13}C NMR spectrum for compound 5

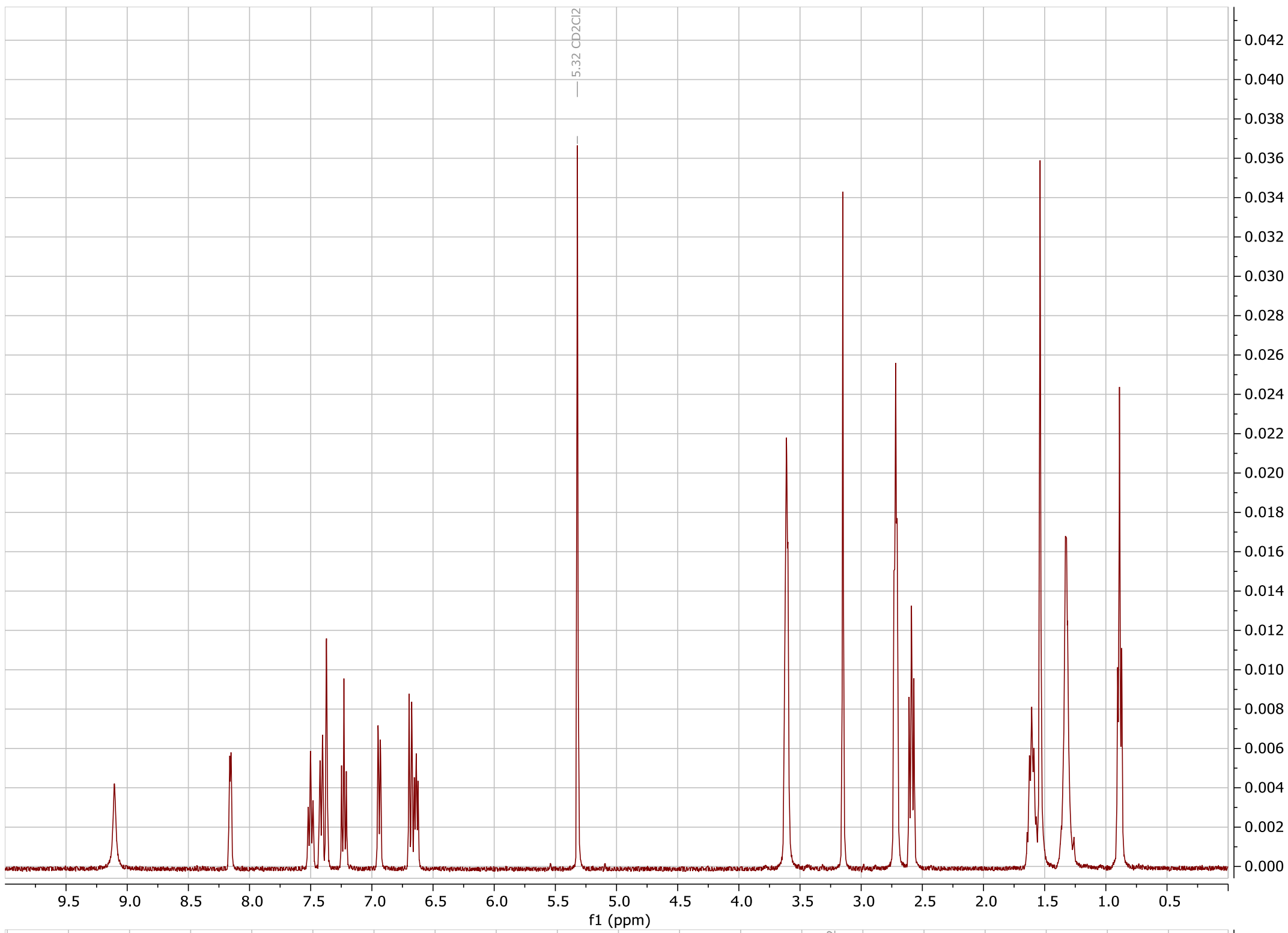

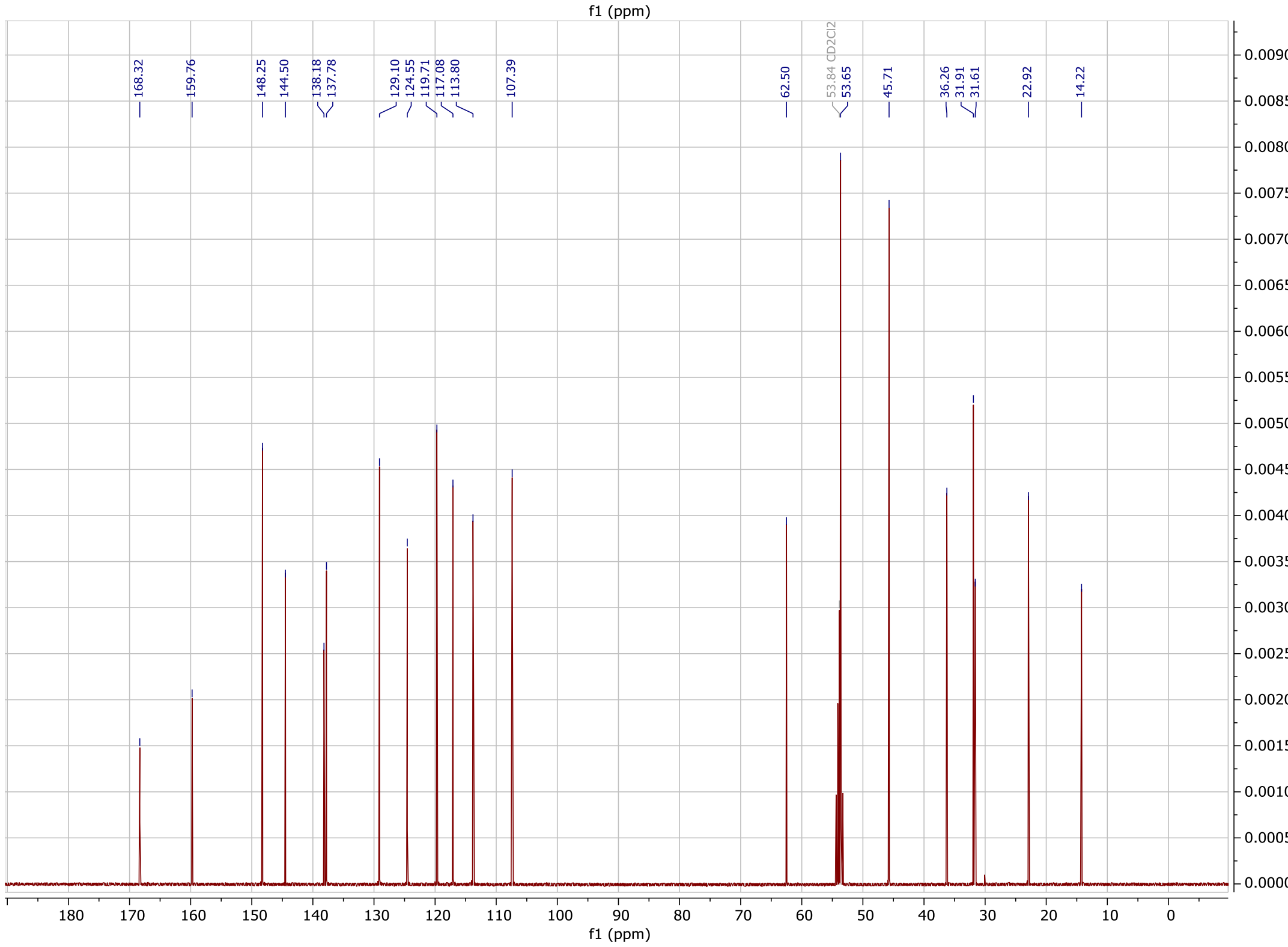

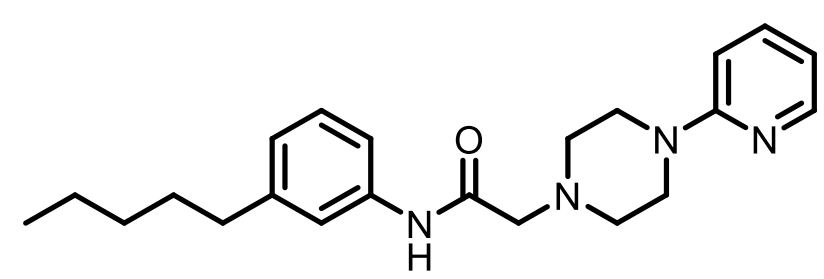

Figure S21. ^{1}H and ^{13}C NMR spectrum for compound 6

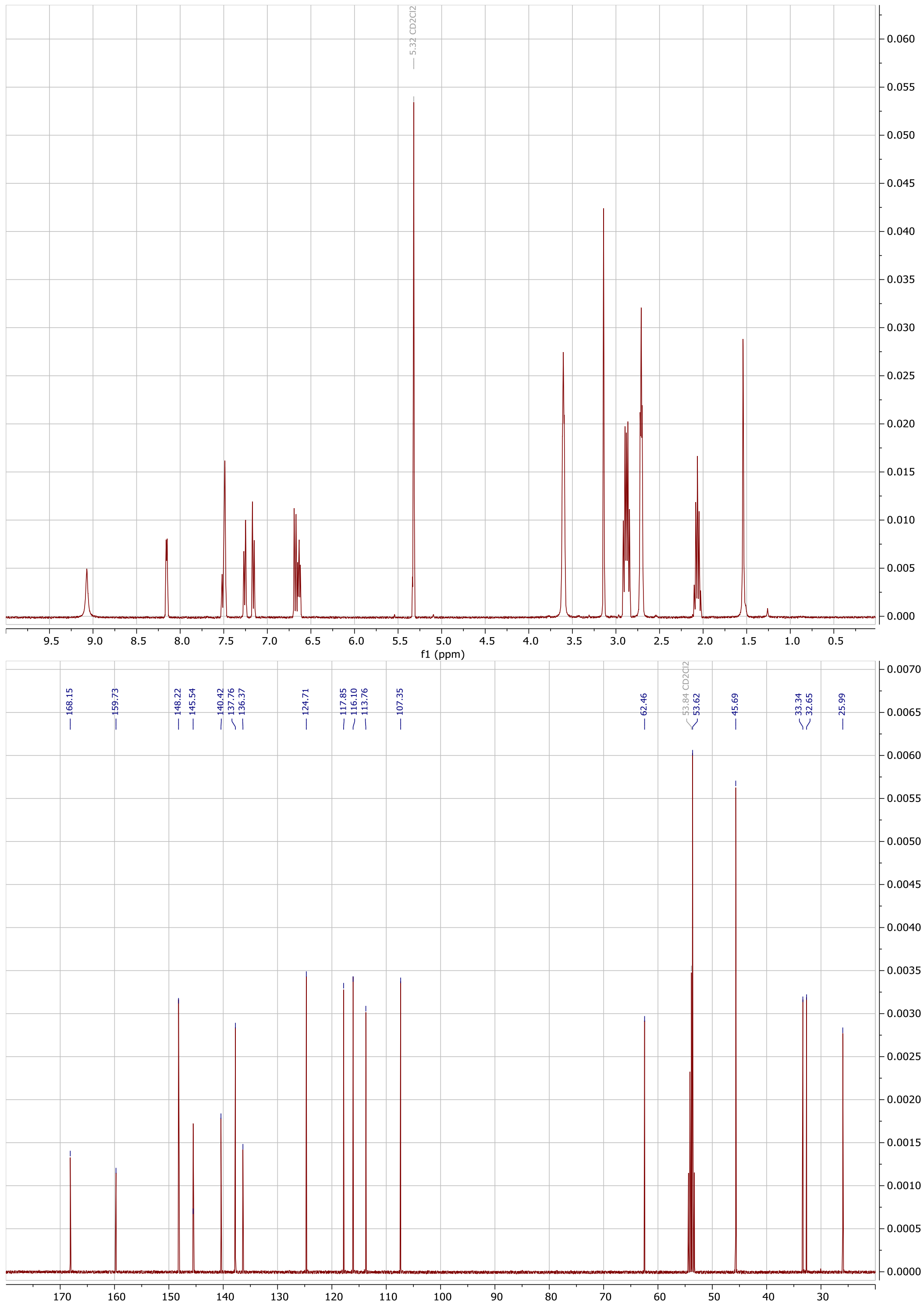

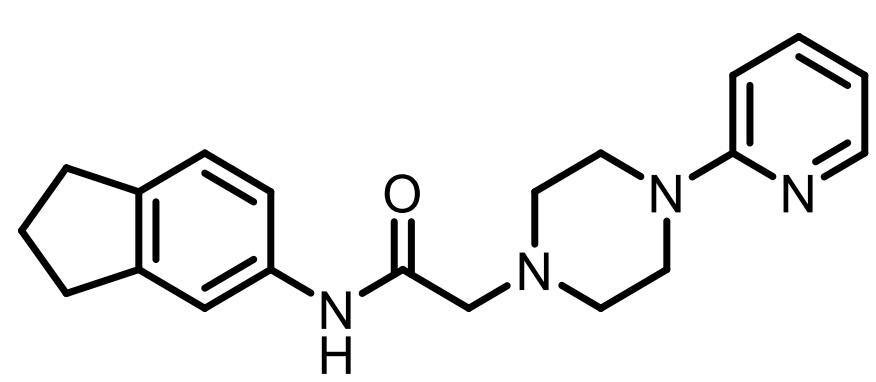

Figure S22. ^{1}H and ^{13}C NMR spectrum for compound 7

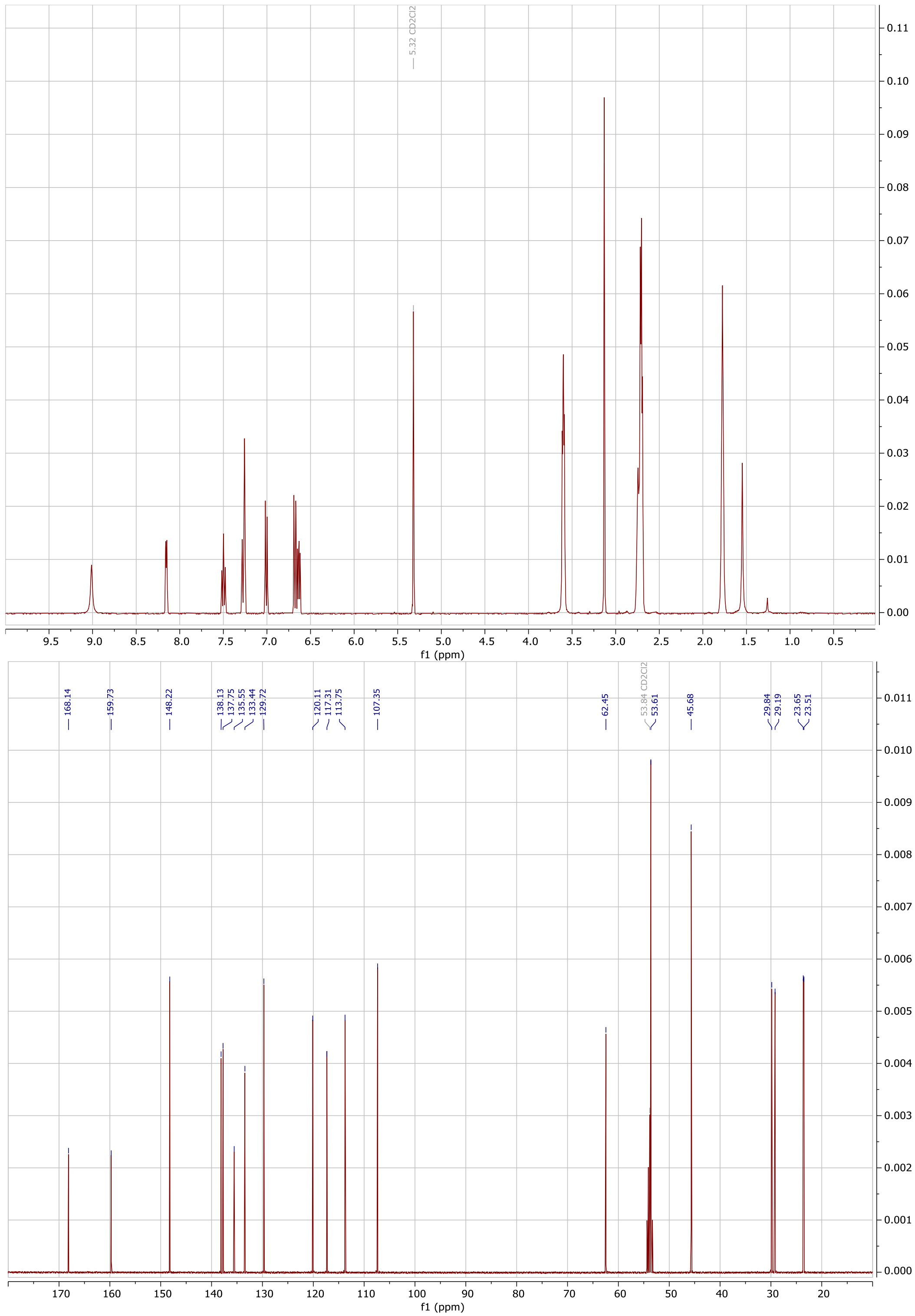

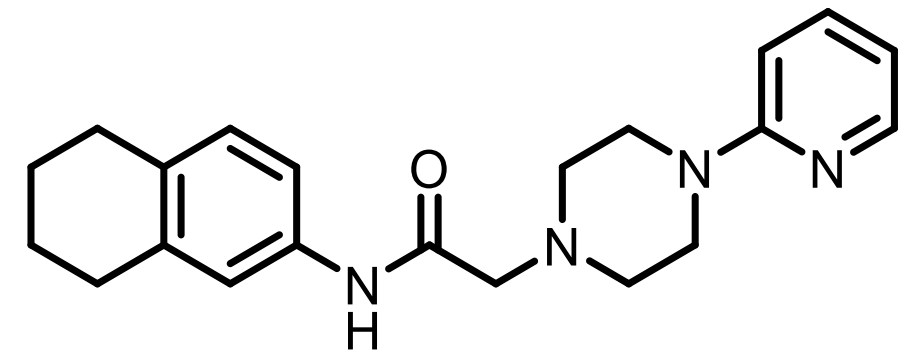

Figure S23. ^{1}H and ^{13}C NMR spectrum for compound 8

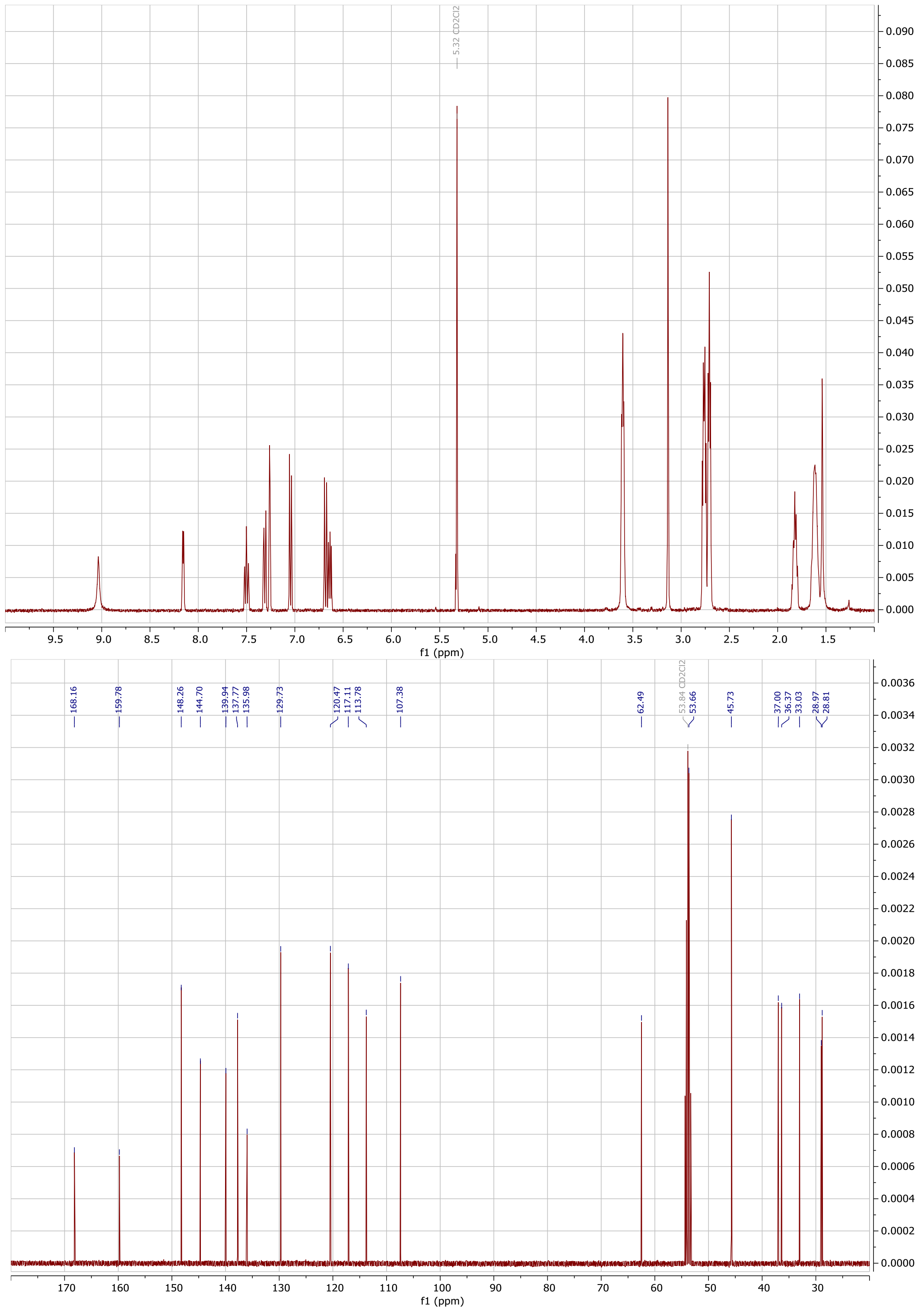

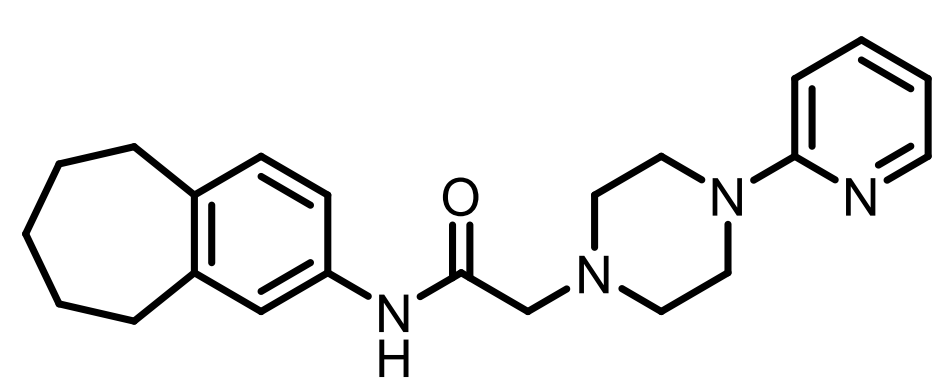

Figure S24. ^{1}H and ^{13}C NMR spectrum for compound 9

References:

1. Wager, T. T.; Hou, X.; Verhoest, P. R.; Villalobos, A. Central nervous system multiparameter optimization desirability: application in drug discovery. *ACS chemical neuroscience* 2016, 7 (6), 767–775.
2. Keck, T. M.; Free, R. B.; Day, M. M.; Brown, S. L.; Maddaluna, M. S.; Fountain, G.; Cooper, C.; Fallon, B.; Holmes, M.; Stang, C. T.; Burkhardt, R.; Bonifazi, A.; Ellenberger, M. P.; Newman, A. H.; Sibley, D. R.; Wu, C.; Boateng, C. A., Dopamine D(4) Receptor-Selective Compounds Reveal Structure-Activity Relationships that Engender Agonist Efficacy. *J Med Chem* **2019,** *62* (7), 3722-3740.
3. Boateng, C. A.; Bakare, O. M.; Zhan, J.; Banala, A. K.; Burzynski, C.; Pommier, E.; Keck, T. M.; Donthamsetti, P.; Javitch, J. A.; Rais, R.; Slusher, B. S.; Xi, Z. X.; Newman, A. H., High Affinity Dopamine D3 Receptor (D3R)-Selective Antagonists Attenuate Heroin Self-Administration in Wild-Type but not D3R Knockout Mice. *J Med Chem* **2015,** *58* (15), 6195-213.
4. Boateng, C. A.; Nilson, A. N.; Placide, R.; Pham, M. L.; Jakobs, F. M.; Boldizsar, N.; McIntosh, S.; Stallings, L. S.; Korankyi, I. V.; Kelshikar, S.; Shah, N.; Panasis, D.; Muccilli, A.; Ladik, M.; Maslonka, B.; McBride, C.; Sanchez, M. X.; Akca, E.; Alkhatib, M.; Saez, J.; Nguyen, C.; Kurtyan, E.; DePierro, J.; Crowthers, R.; Brunt, D.; Bonifazi, A.; Newman, A. H.; Rais, R.; Slusher, B. S.; Free, R. B.; Sibley, D. R.; Stewart, K. D.; Wu, C.; Hemby, S. E.; Keck, T. M., Pharmacology and Therapeutic Potential of Benzothiazole Analogues for Cocaine Use Disorder. *J Med Chem* **2023,** *66* (17), 12141-12162.
5. Cheng, Y.; Prusoff, W. H., Relationship between the inhibition constant (K1) and the concentration of inhibitor which causes 50 per cent inhibition (I50) of an enzymatic reaction. *Biochem Pharmacol* **1973,** *22* (23), 3099-108.
6. Wang, S.; Wacker, D.; Levit, A.; Che, T.; Betz, R. M.; McCorvy, J. D.; Venkatakrishnan, A. J.; Huang, X. P.; Dror, R. O.; Shoichet, B. K.; Roth, B. L., D(4) dopamine receptor high-resolution structures enable the discovery of selective agonists. *Science* **2017,** *358* (6361), 381-386.
7. Zhang, X. C.; Cao, C.; Zhou, Y.; Zhao, Y., Proton transfer-mediated GPCR activation. *Protein Cell* **2015,** *6* (1), 12-7. (b) Schrödinger, L. L. C. Schrödinger Release 2024-4: Maestro. **2024**.
8. Lomize, M. A.; Lomize, A. L.; Pogozheva, I. D.; Mosberg, H. I., OPM: orientations of proteins in membranes database. *Bioinformatics* **2006,** *22* (5), 623-5.
9. Schott-Verdugo, S.; Gohlke, H., PACKMOL-Memgen: A Simple-To-Use, Generalized Workflow for Membrane-Protein-Lipid-Bilayer System Building. *J Chem Inf Model* **2019,** *59* (6), 2522-2528. doi: 10.1021/acs.jcim.9b0026
10. Case, D. A.; Aktulga, H. M.; Belfon, K.; Cerutti, D. S.; Cisneros, G. A.; Cruzeiro, V. W. D.; Forouzesh, N.; Giese, T. J.; Gotz, A. W.; Gohlke, H.; Izadi, S.; Kasavajhala, K.; Kaymak, M. C.; King, E.; Kurtzman, T.; Lee, T. S.; Li, P.; Liu, J.; Luchko, T.; Luo, R.; Manathunga, M.; Machado, M. R.; Nguyen, H. M.; O'Hearn, K. A.; Onufriev, A. V.; Pan, F.; Pantano, S.; Qi, R.; Rahnamoun, A.; Risheh, A.; Schott-Verdugo, S.; Shajan, A.; Swails, J.; Wang, J.; Wei, H.; Wu, X.; Wu, Y.; Zhang, S.; Zhao, S.; Zhu, Q.; Cheatham, T. E., 3rd; Roe, D. R.; Roitberg, A.; Simmerling, C.; York, D. M.; Nagan, M. C.; Merz, K. M.,

Jr., AmberTools. *J Chem Inf Model* **2023,** *63* (20), 6183-6191. doi.org/10.1021/ACS.JCIM.3C01153
11. Case, D. A.; Cheatham, T. E., 3rd; Darden, T.; Gohlke, H.; Luo, R.; Merz, K. M., Jr.; Onufriev, A.; Simmerling, C.; Wang, B.; Woods, R. J., The Amber biomolecular simulation programs. *J Comput Chem* **2005,** *26* (16), 1668-88. doi.org/10.1002/JCC.20290
12. Tian, C.; Kasavajhala, K.; Belfon, K. A. A.; Raguette, L.; Huang, H.; Migues, A. N.; Bickel, J.; Wang, Y.; Pincay, J.; Wu, Q.; Simmerling, C., ff19SB: Amino-Acid-Specific Protein Backbone Parameters Trained against Quantum Mechanics Energy Surfaces in Solution. *J Chem Theory Comput* **2020,** *16* (1), 528-552. doi.org/10.1021/ACS.JCTC.9B00591
13. Dickson, C. J.; Walker, R. C.; Gould, I. R., Lipid21: Complex Lipid Membrane Simulations with AMBER. *J Chem Theory Comput* **2022,** *18* (3), 1726-1736. doi.org/10.1021/ACS.JCTC.1C01217
14. Izadi, S.; Anandakrishnan, R.; Onufriev, A. V., Building Water Models: A Different Approach. *J Phys Chem Lett* **2014,** *5* (21), 3863-3871. doi.org/10.1021/JZ501780A
15. Hopkins, C. W.; Le Grand, S.; Walker, R. C.; Roitberg, A. E., Long-Time-Step Molecular Dynamics through Hydrogen Mass Repartitioning. *J Chem Theory Comput* **2015,** *11* (4), 1864-74. doi.org/10.1021/CT5010406
16. Åqvist, J.; Wennerström, P.; Nervall, M.; Bjelic, S.; Brandsdal, B. O. Molecular Dynamics Simulations of Water and Biomolecules with a Monte Carlo Constant Pressure Algorithm. *Chem Phys Lett* **2004**, 384 (4–6), 288–294. doi.org/10.1063/1.448118
17. Loncharich, R. J.; Brooks, B. R.; Pastor, R. W., Langevin dynamics of peptides: the frictional dependence of isomerization rates of N-acetylalanyl-N'-methylamide. *Biopolymers* **1992,** *32* (5), 523-35. doi.org/10.1002/BIP.360320508
18. Friesner, R. A.; Banks, J. L.; Murphy, R. B.; Halgren, T. A.; Klicic, J. J.; Mainz, D. T.; Repasky, M. P.; Knoll, E. H.; Shelley, M.; Perry, J. K.; Shaw, D. E.; Francis, P.; Shenkin, P. S., Glide: a new approach for rapid, accurate docking and scoring. 1. Method and assessment of docking accuracy. *J Med Chem* **2004,** *47* (7), 1739-49. doi.org/10.1021/JM0306430
19. Halgren, T. A.; Murphy, R. B.; Friesner, R. A.; Beard, H. S.; Frye, L. L.; Pollard, W. T.; Banks, J. L., Glide: a new approach for rapid, accurate docking and scoring. 2. Enrichment factors in database screening. *J Med Chem* **2004,** *47* (7), 1750-9. doi.org/10.1021/JM030644S
20. Friesner, R. A.; Murphy, R. B.; Repasky, M. P.; Frye, L. L.; Greenwood, J. R.; Halgren, T. A.; Sanschagrin, P. C.; Mainz, D. T., Extra precision glide: docking and scoring incorporating a model of hydrophobic enclosure for protein-ligand complexes. *J Med Chem* **2006,** *49* (21), 6177-96. doi.org/10.1021/JM051256O
21. Yang, Y.; Yao, K.; Repasky, M. P.; Leswing, K.; Abel, R.; Shoichet, B. K.; Jerome, S. V., Efficient Exploration of Chemical Space with Docking and Deep Learning. *J Chem Theory Comput* **2021,** *17* (11), 7106-7119. doi.org/10.1103/PhysRev.32.339
22. He, X.; Man, V. H.; Yang, W.; Lee, T. S.; Wang, J., A fast and high-quality charge model for the next generation general AMBER force field. *J Chem Phys* **2020,** *153* (11), 114502. doi.org/10.1063/5.0019056
23. Gotz, A. W.; Williamson, M. J.; Xu, D.; Poole, D.; Le Grand, S.; Walker, R. C., Routine Microsecond Molecular Dynamics Simulations with AMBER on GPUs. 1. Generalized Born. *J Chem Theory Comput* **2012,** *8* (5), 1542-1555. doi.org/10.1021/CT200909J.

24. Salomon-Ferrer, R.; Gotz, A. W.; Poole, D.; Le Grand, S.; Walker, R. C., Routine Microsecond Molecular Dynamics Simulations with AMBER on GPUs. 2. Explicit Solvent Particle Mesh Ewald. *J Chem Theory Comput* **2013,** *9* (9), 3878-88. doi.org/10.1021/CT400314Y
25. Roe, D. R.; Cheatham, T. E., 3rd, PTRAJ and CPPTRAJ: Software for Processing and Analysis of Molecular Dynamics Trajectory Data. *J Chem Theory Comput* **2013,** *9* (7), 3084-95. doi.org/10.1021/CT400341P
26. Meng, E. C.; Goddard, T. D.; Pettersen, E. F.; Couch, G. S.; Pearson, Z. J.; Morris, J. H.; Ferrin, T. E., UCSF ChimeraX: Tools for structure building and analysis. *Protein Sci* **2023,** *32* (11), e4792. doi: 10.1002/pro.4792
27. Li, Y.; Rezaei, M. A.; Li, C.; Li, X. DeepAtom: A Framework for Protein-Ligand Binding Affinity Prediction. Proceedings - 2019 *IEEE International Conference on Bioinformatics and Biomedicine, BIBM* **2019**, 303–310. doi.org/10.1109/BIBM47256.2019.8982964
28. Jin, X.; Han, J. K-Means Clustering. *Encyclopedia of Machine Learning* **2011**, 563–564. doi.org/10.1007/978-0-387-30164-8_425
